# Bacteria-responsive programmed self-activating antibacterial hydrogel to remodel regeneration microenvironment for infected wound healing

**DOI:** 10.1093/nsr/nwae044

**Published:** 2024-01-30

**Authors:** Yutong Yang, Jiaxin Wang, Shengfei Huang, Meng Li, Jueying Chen, Dandan Pei, Zhen Tang, Baolin Guo

**Affiliations:** State Key Laboratory for Mechanical Behavior of Materials and Frontier Institute of Science and Technology, and Key Laboratory of Shaanxi Province for Craniofacial Precision Medicine Research, College of Stomatology, Xi'an Jiaotong University, Xi'an 710049, China; State Key Laboratory for Mechanical Behavior of Materials and Frontier Institute of Science and Technology, and Key Laboratory of Shaanxi Province for Craniofacial Precision Medicine Research, College of Stomatology, Xi'an Jiaotong University, Xi'an 710049, China; State Key Laboratory for Mechanical Behavior of Materials and Frontier Institute of Science and Technology, and Key Laboratory of Shaanxi Province for Craniofacial Precision Medicine Research, College of Stomatology, Xi'an Jiaotong University, Xi'an 710049, China; State Key Laboratory for Mechanical Behavior of Materials and Frontier Institute of Science and Technology, and Key Laboratory of Shaanxi Province for Craniofacial Precision Medicine Research, College of Stomatology, Xi'an Jiaotong University, Xi'an 710049, China; State Key Laboratory for Mechanical Behavior of Materials and Frontier Institute of Science and Technology, and Key Laboratory of Shaanxi Province for Craniofacial Precision Medicine Research, College of Stomatology, Xi'an Jiaotong University, Xi'an 710049, China; State Key Laboratory for Mechanical Behavior of Materials and Frontier Institute of Science and Technology, and Key Laboratory of Shaanxi Province for Craniofacial Precision Medicine Research, College of Stomatology, Xi'an Jiaotong University, Xi'an 710049, China; Department of Orthopedics, Tangdu Hospital, Fourth Military Medical University, Xi'an 710038, China; State Key Laboratory for Mechanical Behavior of Materials and Frontier Institute of Science and Technology, and Key Laboratory of Shaanxi Province for Craniofacial Precision Medicine Research, College of Stomatology, Xi'an Jiaotong University, Xi'an 710049, China; Department of Orthopedics, The First Affiliated Hospital of Xi'an Jiaotong University, Xi'an 710061, China

**Keywords:** bacterial response, hydrogel dressing, multiple nanozyme activity, biofilm elimination, infected motion wound healing

## Abstract

There is still an urgent need to develop hydrogels with intelligent antibacterial ability to achieve on-demand treatment of infected wounds and accelerate wound healing by improving the regeneration microenvironment. We proposed a strategy of hydrogel wound dressing with bacteria-responsive self-activating antibacterial property and multiple nanozyme activities to remodel the regeneration microenvironment in order to significantly promote infected wound healing. Specifically, pH-responsive H_2_O_2_ self-supplying composite nanozyme (MSCO) and pH/enzyme-sensitive bacteria-responsive triblock micelles encapsulated with lactate oxidase (PPEL) were prepared and encapsulated in hydrogels composed of L-arginine-modified chitosan (CA) and phenylboronic acid-modified oxidized dextran (ODP) to form a cascade bacteria-responsive self-activating antibacterial composite hydrogel platform. The hydrogels respond to multifactorial changes of the bacterial metabolic microenvironment to achieve on-demand antibacterial and biofilm eradication through transformation of bacterial metabolites, and chemodynamic therapy enhanced by nanozyme activity in conjunction with self-driven nitric oxide (NO) release. The composite hydrogel showed ‘self-diagnostic’ treatment for changes in the wound microenvironment. Through self-activating antibacterial therapy in the infection stage to self-adaptive oxidative stress relief and angiogenesis in the post-infection stage, it promotes wound closure, accelerates wound collagen deposition and angiogenesis, and completely improves the microenvironment of infected wound regeneration, which provides a new method for the design of intelligent wound dressings.

## Teaser

A self-activated on-demand antibacterial hydrogel based on bacterial infection microenvironment response is proposed for remodeling the regenerative microenvironment of infected motion wounds and accelerating wound healing.

## INTRODUCTION

The worldwide medical burden caused by skin defects has become a major issue that cannot be ignored in the field of biomedicine [1]. The orderly progress of wound healing strictly depends on the close coordination of different healing stages [2]. However, the body's self-protection ability is reduced due to skin damage, which provides an opportunity for bacterial infection. Once bacteria accumulate and colonize in large quantities on the local wound, the body's self-defense mechanism will be activated to recruit inflammatory cells (such as macrophages, neutrophils, etc.), forming an acute inflammatory response [3]. Bacterial infected wounds often form persistent chronic inflammation, which hinders the transition of damaged tissues to the proliferative phase and further delays the healing of infected wounds [4]. In addition, the slow formation of new blood vessels is another characteristic of chronic bacterial infection of wounds. Insufficient angiogenesis will affect the nutrient exchange and oxygen delivery to the wound, which will not be conducive to wound healing [5]. Therefore, in response to the needs of infected chronic wound healing, developing a comprehensive strategy with high-efficiency antibacterial inflammation relief, and accelerated angiogenesis will help the rapid healing of infected wounds [6].

Bacterial infection leads to great changes in the microenvironment around the wound, including an increase in the amounts of certain enzymes secreted by the bacteria and a decrease in the pH of the microenvironment [7]. The enzymes produced by bacterial metabolism include hyaluronidase, gelatinase, lipase and β-lactamase, etc. [8]. These hydrolytic enzymes often cause damage to surrounding healthy tissues and affect the activity of antibiotics [9]. In addition, the residence of bacteria at the infection site will form a biofilm [10], which is conducive to its own anaerobic metabolism to produce acidic substances such as lactic acid, malic acid and acetic acid [11], and reduce the pH value of the wound microenvironment, in particular the pH of the wound microenvironment caused by methicillin-resistant *Staphylococcus aureus* (MRSA) infection may decrease to about 5.5 or even lower [12]. In fact, bacteria that persist under antibiotic pressure are often the culprits for the development of drug resistance and recurrence of infection, while conventional antibiotic treatment fails to effectively disrupt biofilms [13]. Therefore, based on the complex chemical, physical and biological properties of bacterial infection of wounds, the antibiotic treatment of bacterial infections faces major challenges, and it is urgent to explore innovative strategies to inhibit drug-resistant bacterial infections.

At present, emerging therapeutic methods (such as photothermal therapy, photodynamic therapy, chemodynamic therapy, and gas therapy, etc.) have been used to treat bacterially infected wounds and have achieved satisfactory results. However, these approaches have relatively low bioavailability and limited efficacy. Stimuli-responsive biomaterials have been developed for the treatment of drug-resistant bacteria infected wounds [14]. Under the action of external or internal stimuli, stimuli-responsive biomaterials can realize on-demand antibacterial effects by releasing bactericidal components or changing their own physicochemical properties [15], thus showing great application prospects in the future. Although external stimuli are more controllable than internal stimuli, these treatments are often applied after a serious infection has developed, adding to the suffering of the patient [16]. Endogenous stimuli can respond to changes in the wound microenvironment in a timely manner and achieve maximum suppression of the infection [17]. In addition, the microenvironment of the bacterial infection is often accompanied by changes in multiple factors [18]. In the process of bacterial infection, multiple stimuli-responsive wound dressings, including pH-sensitive and bacterial metabolic enzyme-sensitive, can autonomously exert antibacterial effects only when infection occurs [19]. In view of the multifactorial characteristics of bacterial infection, there is an urgent need to develop intelligent self-activating on-demand antibacterial wound dressings that respond to the bacterial microenvironment in order to achieve self-identification and self-treatment.

**Figure 1.**
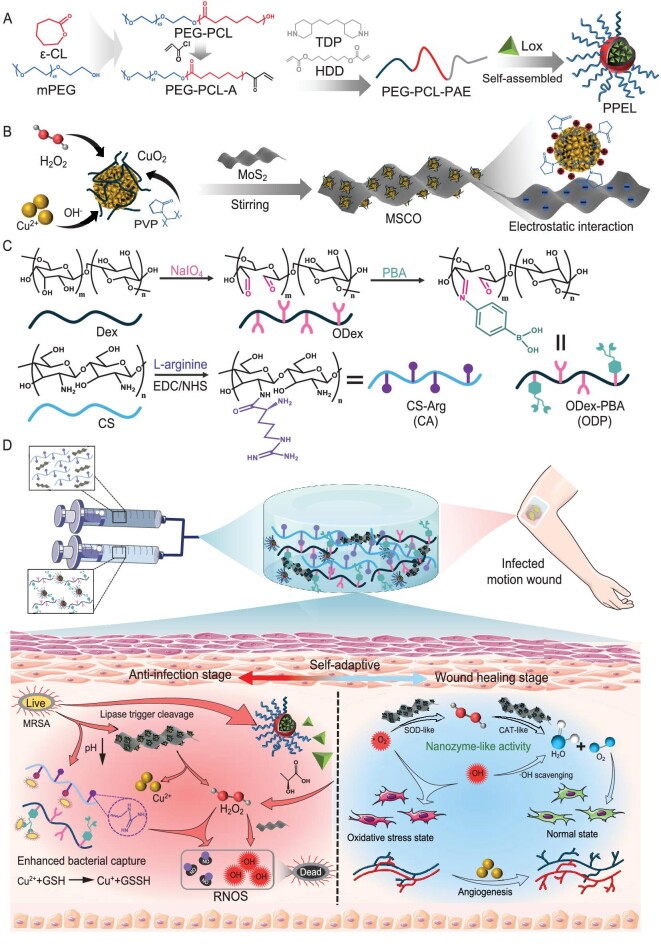
Schematic illustration of the preparation and application of multiple bacteria-responsive hydrogel. (A) Synthesis of Lox- loaded PPE micelle (PPEL). (B) Synthesis of MSCO nanozyme. (C) Synthesis of ODex-PBA (ODP) and CS-Arg (CA). (D) Self-adaptive treatment mechanism of hydrogel for infected motion wound healing.

Here, a series of smart self-activating on-demand antibacterial hydrogels with response to the microenvironment of bacterial infection were designed and fabricated, which have the capability to remodel the regeneration microenvironment for the treatment of infected motion wounds (Fig. 1). First, we synthesized polyethylene glycol-polycaprolactone-poly-β-aminoester triblock copolymer micelles (PEG-PCL-PAE) (PPE) with pH/lipase response for encapsulating lactate oxidase (Fig. 1A). Second, pH-responsive H_2_O_2_ self-supply chemodynamic therapy donor CuO_2_ nanoparticles were synthesized and anchored on the surface of MoS_2_ nanosheets to form a composite nanozyme (MSCO) through electrostatic interactions (Fig. 1B). L-arginine modified chitosan (CA) and phenylboronic acid modified oxidized dextran (ODP) (Fig. 1C) constituted self-healing hydrogels through dynamic Schiff base bonds and phenylboronate bonds, and achieved self-identification and self-treatment of infection by encapsulating MSCO nanozyme as well as Lox- loaded PPE micelles (PPEL). Specifically, in the acidic microenvironment of bacterial infection, the network structure of the hydrogel collapsed to release the PPEL micelle and MSCO nanozyme. PPEL released the loaded Lox in the presence of acidic metabolites and lipase. Lox catalyzed the decomposition of lactic acid to produce H_2_O_2_, and cooperated with the H_2_O_2_ hydrolyzed by the MSCO nanozyme to catalyze the production of nitric oxide (NO) from L-arginine of CA. In the presence of H_2_O_2_, the peroxidase-like (POD-like) activity of MoS_2_ enhanced the Cu^2+^ catalyzed Fenton reaction produced ROS, effectively eliminating bacteria with the help of NO. Furthermore, MSCO exerted GSH elimination activity through the released Cu^2+^ further weakening the ROS defense ability of bacteria. After the infection was eliminated, the regeneration microenvironment should be remodeled to enhance wound healing. The hydrogel effectively relieved oxidative stress by exerting multiple nanozyme activities in weakly alkaline wound microenvironments, and the Cu^2+^ released through the slow hydrolysis of MSCO cooperated with L-arginine to promote wound angiogenesis (Fig. 1D). Cu^2+^ promote blood vessel formation by regulating vascular endothelial cell growth factor (VEGF) [20]. Overall, the constructed composite hydrogel possessed intelligent bacteria-responsive self-activating on-demand antibacterial ability, promoting relief of oxidative stress and vascularization of regenerative tissue, which is an effective strategy to realize the healing of infected motion wounds.

## RESULTS AND DISCUSSION

### Synthesis and characterization of MSCO nanozyme and PPEL micelle

**Figure 2.**
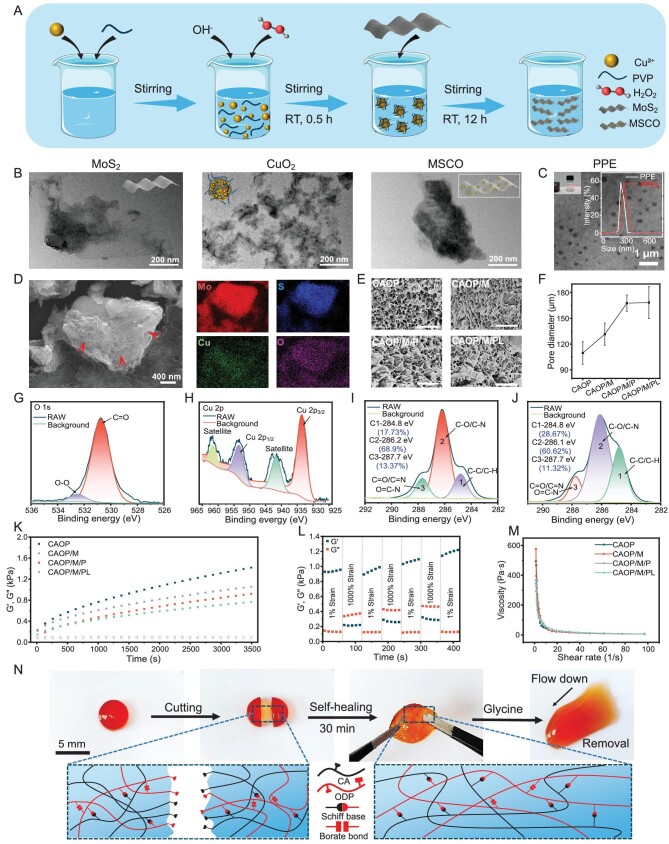
(A) Schematic illustration of the synthesis of the MSCO nanozyme. TEM images of (B) MoS_2_ nanosheets, CuO_2_ nanoparticles and MSCO nanozyme and (C) PPE micelles, Tyndall effect of PPE micelles and size change of micelles after loading Lox. (D) SEM image and element mapping of MSCO nanozyme. (E) SEM images of hydrogel, scale bar: 500 μm. (F) Pore diameter of hydrogels. High-resolution XPS spectra of (G) O 1s, (H) Cu 2p, (I) CAOP hydrogel, and (J) CAOP/M/PL hydrogel. (K) Rheological properties of hydrogels. (L) Self-healing property of CAOP/M/PL. (M) Shear-thinning properties of the hydrogel. (N) Macroscopic self-healing presentation, mechanisms and on-demand removal capability of hydrogel.

Inspired by the microenvironmental characteristics of bacterial infection, a self-assembled nanozyme with amplified cascading antibacterial effects in response to pH changes was synthesized. Following the synthesis process in Fig. 2A, the CuO_2_ coated MoS_2_ nanozyme (MSCO) was synthesized by simple electrostatic self-assembly. The morphology changes of the nanozyme were evaluated by TEM. Compared with the structure of MoS_2_ nanosheets, the structure of MSCO nanozyme becomes rough after being coated by CuO_2_ (Fig. 2B). The SEM image of MSCO further verified that CuO_2_ was coated on the surface of MoS_2_ (red arrow) and element mapping confirmed the uniform distribution of Cu element (green) and O element (purple) (Fig. 2D). To confirm that the self-assembly mechanism of the MSCO nanozyme was derived from electrostatic force, the surface charge changes of the nanomaterials were evaluated (Fig. S1). Zeta potential indicated the surface potential of MoS_2_ changes from negative to positive after being coated by CuO_2_. AFM results indicated that the thickness of the MSCO nanozyme was about 30.7–37.9 nm (Fig. S2). Furthermore, the XRD results of MSCO show that the modification of CuO_2_ will not affect the crystal structure of MoS_2_ itself (Fig. S3). In summary, CuO_2_ coated MoS_2_ nanozymes were successfully prepared by electrostatic self-assembly. Meanwhile, the presence of peroxy groups in the nanozyme was verified by potassium permanganate colorimetry (Fig. S4) [21]. The chemical structure composition of the MSCO nanozyme was analyzed by XPS spectrum. Two O 1s peaks at 530.7 and 532.5 eV were assigned to C=O and O−O, respectively, suggesting the existence of PVP and peroxy groups (Fig. 2G). The Cu 2p XPS spectrum displayed two main peaks at 933.6 and 953.5 eV accompanied by two satellite peaks at 941.7 and 961.8 eV, respectively, which indicated the valence state of Cu in MSCO nanozyme was +2 (Fig. 2H). Obvious N 1s, O 1s and Cu 2p3 peaks were observed in the full XPS spectrum of MSCO, indicating the presence of PVP and CuO_2_ in MSCO (Fig. S5).

Amphiphilic triblock copolymer PEG-PCL-PAE (PPE) was sensitivity to pH and enzyme signals [22], which may serve as stimuli-responsive release vehicles. First, diblock copolymer PEG-PCL was synthesized by the ring-opening reaction of caprolactone (CL) with methoxy terminated PEG (mPEG). ^1^H NMR spectrum confirmed the structure of PEG-PCL (Fig. S6), PEG-PCL-acrylate (PEG-PCL-A) (Fig. S7) and PEG-PCL-PAE (PPE) (Fig. S8). The PPE can be self-assembled into micelles by the emulsion/solvent evaporation method. TEM imaging of PPE micelles showed a well-defined spherical structure (Fig. 2C). After loading lactate oxidase (Lox), the size of the PPEL micelles slighted increased (Fig. 2C inset and Fig. S9A), and the surface negative charge was reduced compared to PPE micelle (Fig. S9B). In addition, the encapsulation efficiency of Lox by PPE micelles was estimated to be about 51.4% by bicinchoninic acid (BCA) assay. The applicability of PPE micelles under bacterial infection conditions was explored. PPE exhibited a surface charge transition behavior that adapts to changes in the pH of the surrounding environment (Fig. S10). Based on the lipase-sensitive characteristics of PCL and the acidic hydrolysis characteristics of ester bonds [9], the hydrophilic/hydrophobic balance of the micellar structure was destroyed, resulting in a significant change in its morphology (Fig. S11), which indicated that any one of the above two stimuli could cause the disruption of the micellar structure and release the contents.

### Synthesis and characterization of composite hydrogels

Considering the needs of motion infected wound healing, self-healing hydrogels based on dynamic Schiff base bonds and phenylboronate bond were prepared by L-arginine modified chitosan (CA) and phenylboronic acid modified oxidized dextran (ODP). FT-IR spectrum confirmed the structure of CA and ODP (Fig. S12) [23]. The chemical structures of CA and ODP were also confirmed by ^1^H NMR (Figs S13, S14) [24,25].

To explore the need for hydrogels with both mechanical properties and biocompatibility, the effect of polymer concentrations on hydrogel adhesion strength and hemolysis were evaluated (Figs S15, S16). Considering the needs of motion wound healing, the hydrogel with 1.5 wt% CA and 2.0 wt% ODP was chosen for subsequent studies (discussion in Supplementary Data) and named it CAOP. In addition, the CAOP loaded with MSCO was named as CAOP/M; the CAOP loaded with MSCO and PPE was named as CAOP/M/P; when the PPE was loaded with Lox (PPEL), the CAOP loaded with MSCO and PPEL was named as CAOP/M/PL.

The chemical structure changes of hydrogel were analyzed by XPS spectrum (Fig. 2I and J). The polysaccharide-based hydrogel contained a large amount of C 2 (C–O), which was consistent with their structures. The C 1s spectrum of CAOP/M/PL showed decreased C 3 (C=N) content and increased C 1 (C–C) content compared to CAOP, which was attributed to the interference of MSCO and PPE with the hydrogel chemical bond crosslinking. Meanwhile, PVP and PPE may be the main reasons for the increase of C 1 content in the CAOP/M/PL.

The changes in the pore size structure of the hydrogel were explored (Fig. 2E and F). CAOP exhibited the densest pore structure. The doping of MSCO and PPEL increased the pore size of the hydrogel from 109.6 μm to 168.5 μm, which confirmed a decrease in the cross-linking density of the hydrogel.

### Mechanical behavior, swelling, degradation and adhesion property

The storage modulus changes of the hydrogels were evaluated by rheological characterization (Fig. 2K). CAOP has the highest storage modulus, indicating it has the highest degree of crosslinking, which is consistent with the results observed by SEM. With the doping of nanozyme and micelle, the storage modulus of the hydrogel gradually decreased, which was attributed to the disturbance of the chemical bond crosslinking by the two nanocomponents. The critical strain point at which the hydrogel network collapses was determined through strain amplitude scanning testing (Fig. S17). The self-healing property of CAOP/M/PL was subsequently evaluated (Fig. 2L). Under alternating shear strains, the network structure of the hydrogel underwent collapse and rapid recovery, and did not show significant modulus loss, indicating that CAOP/M/PL has good self-healing performance and has potential for motion wound healing. In addition, all hydrogels also exhibited good shear-thinning ability (Fig. 2M). The macroscopic self-healing ability of the hydrogel was further evaluated (Fig. 2N). After the hydrogel block was cut and allowed to heal at 37°C for 30 min, the self-healed hydrogel could withstand a large degree of deformation without fracture. Furthermore, the hydrogel undergoes a gel-sol transition in the presence of glycine, which demonstrated the on-demand removal capability for the painless replacement of wound dressings.

The macroscopic shear thinning test of the hydrogel showed that it can be applied to the target site through a simple injection device, and the hydrogel at the target site can undergo stretching, twisting and squeezing without falling off and breaking, which further proved that hydrogel was able to provide stable protection and connection to the wound (Fig. S18A). The stability performance of the hydrogel in finger joints after frequent activities was also evaluated (Fig. S18B). The results showed that the hydrogel can still maintain a tight connection to the fitting site after being twisted at different angles and washed by water. The tensile properties of the hydrogel were further evaluated (Fig. S18C). The CAOP showed the largest elongation at break and peak stress. With the doping of composite nanozymes and micelles, the elongation at break and peak stress of the hydrogel decreased significantly. In addition, the strip shaped hydrogel can also sequentially recover to the initial state after undergoing different degrees of torsion (Fig. S18D and Movie 1). The adhesion performance of the hydrogel to porcine skin was evaluated (Fig. S18E), CAOP had the highest adhesion strength (about 16.18 kPa), while the adhesion strength of CAOP/M, CAOP/M/P, and CAOP/M/PL decreased to 12.50 kPa, 11.06 kPa, and 11.08 kPa, respectively. The sealing ability of the hydrogel was assessed using cracked water bottles filled with water. When the hydrogel sealed the damaged bottle, no liquid leakage occurred from the water-filled bottle, which indicated that the hydrogel had good adhesion and toughness (Fig. S18F). The adhesion mechanism of the hydrogel is shown in Fig. S18G. The force that mediated the tight adhesion between hydrogel and tissue included physical and chemical effects, in which the aldehyde group and phenylboronic acid can form a strong chemical bond with the amino group and hydroxyl group in the tissue [26], amino group and positively charged guanidine group can form hydrogen and ionic bonds with thiol groups and carboxyl groups on the skin surface [27]. The good hemostatic effect of the hydrogel was confirmed duo to the hydrogel's excellent tissue adhesion (Figs S19, S20).

The swelling and degradation performance of the hydrogel was evaluated under a simulated physiological environment (Fig. S18H, S18I). All the hydrogels almost reached swelling equilibrium after 12 h, among which CAOP/M/P and CAOP/M/PL showed a higher degree of swelling, and CAOP showed the lowest swelling ratio due to its denser degree of crosslinking. In addition, the hydrogel exhibited continuous degradation behavior within 250 h, and the hydrogel had a faster degradation rate as the cross-linking degree of the polymer decreased.

### Cascade reaction of stimuli-responsive release

**Figure 3.**
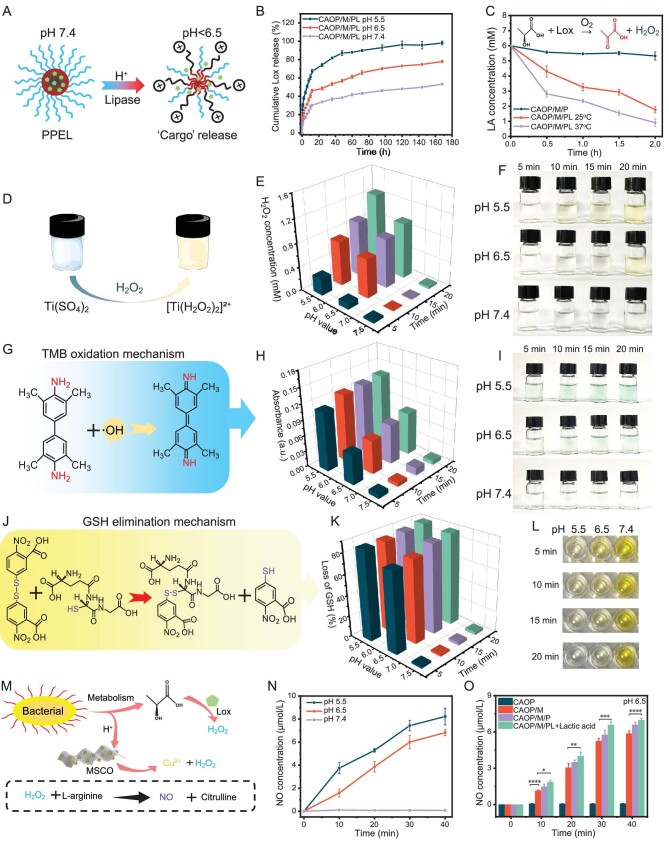
(A) Mechanism of pH/lipase-responsive ‘cargo’ release of PPEL micelle. (B) Cumulative Lox release performance of CAOP/M/PL at different pH. (C) Scavenging behavior of hydrogels towards 6 mM lactic acid (LA) in the presence of lipase. (D) Schematic illustration of the color change of Ti(SO_4_)_2_ in the presence of H_2_O_2_. (E) pH-responsive self-supply H_2_O_2_ behavior of CAOP/M/PL. (F) Color change of Ti(SO_4_)_2_ solution under different treatments.(G) Mechanism of TMB oxidation under **·**OH presence. (H) pH-responsive **·**OH generation of CAOP/M/PL. (I) Color change of TMB solution under different treatments. (J) GSH elimination mechanism. (K) Loss of GSH treated by CAOP/M/PL under different conditions. (L) Color changes of GSH under different treatments. (M) Mechanism of bacteria-responsive NO cascade release. (N) Concentration of NO release treated by CAOP/M/PL under different pH. (O) Cumulative NO release performance of hydrogels at pH 6.5. (*P < 0.05, **P < 0.01, ***P < 0.001, ****P < 0.0001).

Cascading self-activating antibacterial hydrogels triggered by multiple factors based on the bacterial microenvironment were designed to respond to characteristic factors of bacterial infection, thereby exhibiting on-demand antibacterial behavior. Lactic acid is the main reason for the decrease of pH in the wound microenvironment during bacterial metabolism, Lactate oxidase (Lox) produces pyruvate and H_2_O_2_ in the process of oxidizing lactic acid [28], which will be used as a novel means to generate ROS by using the bacteria's own metabolites [29]. However, based on the need to achieve the stimuli-responsive on-demand release of lactate oxidase, we expect to construct a micelle carrier encapsulated in hydrogel that is stable in a physiological environment and releases ‘cargo’ during bacterial infection, triggering the release of lactate oxidase by acid- or enzyme-induced micelle disintegration (Fig. 3A). To verify the stimuli-responsive release characteristics of PPEL micelles encapsulated in hydrogel, the pH-responsive Lox release performance of CAOP/M/PL was tested (Fig. 3B). The Lox release from CAOP/M/PL reached 53.2% within 168 h under pH 7.4, and as the pH decreased to 6.5 and 5.5, the Lox release was reached at 77.9% and 98.1%, respectively, indicating that the acidic environment can accelerate the release of Lox. Subsequently, the ability of the hydrogel to decompose lactic acid in the presence of lipase was further evaluated (Fig. 3C). It should be noted that the acidic environment of the lactic acid solution itself causes the Schiff base bond of the hydrogel to break [30], which in turn causes the PPEL micelle to release Lox in the lipase and acidic environment. The initial content of lactic acid was decomposed by Lox from 6 mM to 1.78 mM after 2 h at 25°C, the decomposition of lactic acid was intensified due to the increased activity of Lox at 37°C, resulting in obvious decrease in the content of lactic acid to 0.92 mM. Not surprisingly, CAOP/M/P could hardly induce the decrease of lactate content, which confirmed that the decomposition of lactate was caused by Lox released from the hydrogel [31].

Subsequently, the H_2_O_2_ self-supply behavior of the CAOP/M/PL under different pH conditions was verified, and using Ti(SO_4_)_2_ as a H_2_O_2_ indicator (Fig. 3D) [32]. According to the linear relationship between the absorbance of Ti(SO_4_)_2_ solution at 410 nm wavelength and various concentrations of H_2_O_2_ (Fig. S21), ∼1.26 mM H_2_O_2_ could be obtained from the CAOP/M/PL at pH 5.5 for 20 min (Fig. 3E), which had a maximum efficiency in comparison to the other conditions, and corresponding Ti(SO_4_)_2_ solution exhibited the most obvious color change (Fig. 3F). In summary, the self-supplying H_2_O_2_ ability of hydrogel depends on the Lox-catalyzed decomposition of lactic acid and the hydrolysis of MSCO nanozyme. It also should be noted that this H_2_O_2_ self-supply phenomenon was highly dependent on the pH changes of the microenvironment.

As an ROS species with high antibacterial efficiency, **·**OH has higher antibacterial ability than the H_2_O_2_ [33]. Based on the above verified pH-responsive H_2_O_2_ self-supply characteristics of hydrogels, we speculate CAOP/M/PL may be proposed as an effective **·**OH source via the Fenton-type reaction between Cu^2+^ and self-supplied H_2_O_2_ and the peroxidase-like activity (POD-like activity) of MoS_2_ under acidic conditions [34,35]. The generated **·**OH was verified by the color change after 3,3′,5,5′-tetramethyl-benzidine (TMB) oxidation (Fig. 3G). The oxidation performance of MSCO nanozyme on TMB at different pH was first evaluated (Fig. S22). Only CuO_2_ nanoparticles and MSCO nanozyme at pH 5.5 exhibited an obvious color transition after co-incubation with TMB. Surprisingly, the MSCO nanozyme showed enhanced TMB oxidation behavior compared to CuO_2_ nanoparticles, which was attributed to the POD-like activity of MoS_2_. Subsequently, the **·**OH production capacity of CAOP/M/PL at different pH was also evaluated (Fig. 3H). Similar to the trend of H_2_O_2_ production, the **·**OH production capacity of hydrogel showed obvious pH dependence, which also verified that H_2_O_2_ was the main source of **·**OH production. As expected, the CAOP/M/PL led to the most obvious color change of TMB under pH 5.5 (Fig. 3I).

Glutathione (GSH) is a key antioxidant system component for bacteria to inhibit their own oxidative stress. Although it has been confirmed that the **·**OH produced by the hydrogel through H_2_O_2_ has potential antibacterial ability, it is still necessary to further evaluate the damage ability of these ROS to the bacterial antioxidant system. The GSH elimination activity of the hydrogel was confirmed. GSH was oxidized to colorless oxidized glutathione (GSSH), causing the solution color to change from yellow to colorless (Fig. 3J). As shown in Fig. 3K, as the pH decreased, the level of GSH decreased following the catalytic reaction between Cu^2+^ released from MSCO nanozyme and GSH to generate Cu^+^ and GSSG [36]. CAOP/M/PL exhibited the most Cu^2+^ release (∼15.4 μM/L) after 48 h under pH 5.5 (Fig. S23). Not surprisingly, CAOP/M/PL caused the most obvious color change of GSH at pH 5.5, indicating almost complete elimination of GSH (Fig. 3L). The robust GSH elimination ability of hydrogel helps to reduce the quenching of ROS. Meanwhile, we also excluded the possible effect of pH on GSH loss, the loss of GSH under acidic conditions was not significantly different from that at pH 7.4 (Fig. S24). Based on the above research results, we confirmed that the hydrogel exhibited good GSH elimination activity and had the potential to destroy the bacterial antioxidant defense system.

NO has shown great potential in the repair of infected wounds because it reacts with ROS to generate reactive nitrogen oxides (RNOSs) with high antibacterial activity [37]. However, most of the reported cases of NO in the repair of infected wounds focus on the release of NO triggered by the external environment [38–40], while the cases of self-driven NO release triggered by the infected microenvironment were rarely reported. For this reason, we speculated whether it is possible to achieve self-driven NO release by changing the pH value of the environment around the hydrogel to mimic the changes in the wound microenvironment after bacterial infection (Fig. 3M). The NaNO_2_ standard curve was used as a reference (Fig. S25). CAOP/M/PL hardly releases NO within 40 min under pH 7.4, which indicated that it has good stability in the physiological environment; while the NO release content of CAOP/M/PL increased from 6.8 μM/L to 8.2 μM/L as the pH decreased from 6.5 to 5.5, which confirmed our speculation that NO release was caused by H_2_O_2_ (Fig. 3N). In addition, the NO release performance of the hydrogel at pH 6.5 was further evaluated (Fig. 3O). Except for CAOP, the NO release from the hydrogels gradually increased over 40 min. Compared with CAOP/M, the NO release of CAOP/M/PL was increased after 40 min (*P* < 0.0001), which was attributed to the oxidation of L-arginine driven by H_2_O_2_ generated from the decomposition of lactic acid catalyzed by Lox. In summary, this intelligent release of NO could respond to the bacterial infection environment, which is expected to enhance ROS-mediated self-activating antibacterials through changes in the degree of wound infection and respond in time to early infection.

### Self-activating antibacterial capability

**Figure 4.**
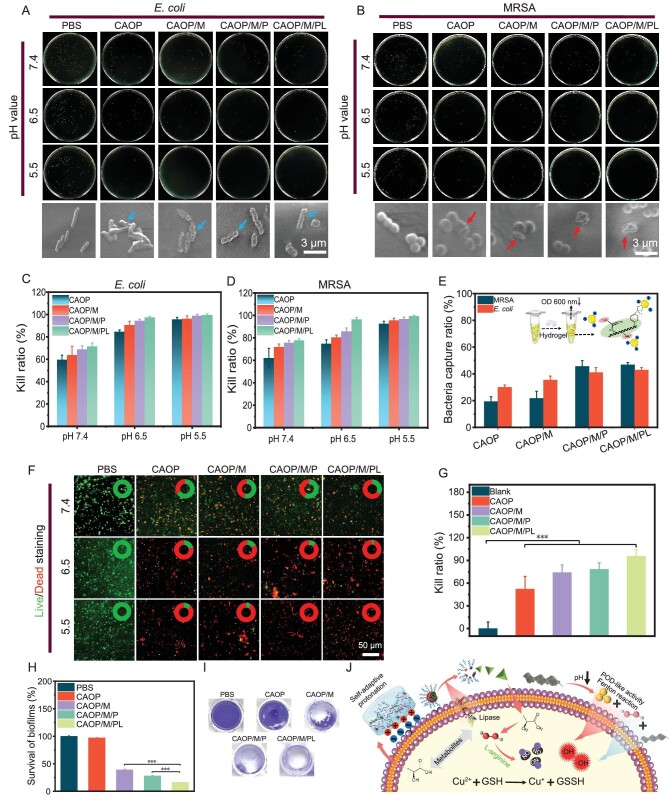
The antibacterial effect presentation and SEM morphology changes of hydrogels against (A) *E. coli* and (B) MRSA. Antibacterial ratio of hydrogels against (C) *E. coli* and (D) MRSA at different pH. (E) Bacteria capture ability of hydrogels for *E. coli* and MRSA. (F) Live/dead staining images of MRSA under different treatments. (G) Kill ratio of *in vivo* antibacterial test. (H) Survival of biofilms after different treatments. (I) Crystal violet staining of surviving biofilms. (J) Schematic illustration of the antibacterial mechanism. (***P < 0.001).

Based on the enhanced CDT effect of self-driven cascade NO delivery synergistically POD-like activity triggered by the bacterial microenvironment, the *in vitro* antibacterial properties of the hydrogel were evaluated. *Escherichia coli* (*E. coli*) and methicillin-resistant *S. aureus* (MRSA) were used for antibacterial testing. The antibacterial efficiency of the hydrogel against *E. coli* at different pH was first evaluated (Fig. 4A and C). All hydrogels exhibited an antibacterial efficiency higher than 59% under pH 7.4, which may be attributed to the positively charged guanidinium and amino groups of CA destroying the bacterial structure through electrostatic forces [41]. In addition, the antibacterial efficiency of the hydrogel increased with the decrease of the pH, and the antibacterial efficiency of CAOP/M/PL reached 98.2% and 99.4% at pH 6.5 and pH 5.5, respectively. Consistent with the quantitative results, the morphology changes of *E. coli* after different treatments at pH 5.5 were observed by SEM (blue arrow in Fig. 4A; the bacterial structure after hydrogel treatment showed obvious deformation, collapse and fragmentation. The antibacterial effect of the hydrogel against MRSA was further explored (Fig. 4B and D). Consistent with the above results, the antibacterial effect of the hydrogel against MRSA also showed a significant pH dependence, in which the antibacterial efficiencies of CAOP/M/PL at pH 7.4, 6.5, and 5.5 were 77.6%, 96.4%, and 99.2%, respectively. Similarly, the SEM morphology of MRSA showed a gradually ruptured and collapsed bacterial surface structure (Fig. 4B red arrow). Live/dead staining was also used to assess MRSA survival after hydrogel treatment (Fig. 4F). The inhibitory effect of the three PBS on bacteria is almost negligible (green), while the proportion of dead bacteria (red) increased after hydrogel treatment (Fig. S26). When the pH was lowered to 5.5, there were almost no obvious living bacteria, which was also consistent with the conclusion of the quantitative results.

The presence of special chemical groups on the surface of the hydrogel exhibits a strong affinity for bacteria [42]. Therefore, the ability of the hydrogel to capture MRSA and *E. coli* at pH 6.5 was evaluated (Fig. 4E). The capture efficiencies of CAOP for MRSA and *E. coli* were 19.3% and 30%, respectively, while the capture efficiencies of CAOP/M/PL for MRSA and *E. coli* increased to 46.8% and 42.9%, respectively, which may be attributed to more free amino and phenylboronic acid groups exposed on the surface of the hydrogel for bacterial capture. The mechanism of the bacteria capture property of the hydrogel can be attributed to the electrostatic interaction between the protonated amino and guanidine groups and the negatively charged bacterial cell wall [43], while phenylboronic acid formed a stable borate bond with the peptidoglycan structure of the bacterial cell wall [44], realizing the chemical combination of bacteria (Fig. 4E inset).

The bacterial biofilm formed by drug-resistant bacteria becomes an obstacle to antibiotic treatment, and the ability of the hydrogel to remove the biofilm under the acidic condition of bacterial infection (pH 6.5) was evaluated by establishing the MRSA biofilm (Fig. 4H and I). Based on the good permeability of NO to the biofilm, CAOP/M/PL showed the best removal effect, and its biofilm residual ratio was 16.4%, which was significantly better than that of CAOP/M (28.0%) and CAOP/M/P (39.1%) (*P* < 0.001). In addition, the biofilm residues after CAOP treatment were not significantly different from PBS, which was attributed to its lack of self-supplying H_2_O_2_ properties and thus unable to trigger the self-activated release of NO.

The *in vivo* antibacterial ability of the hydrogel was further evaluated by establishing a mouse subcutaneous infection model (Fig. 4G). The infected tissues after different treatments were homogenized and inoculated in agarose culture dishes to observe the number of colonies. Compared with the blank, the number of colonies after hydrogel treatment was significantly reduced (*P* < 0.001). Among them, the CAOP/M/PL group showed the best antibacterial effect with no obvious colonies (Fig. S27).

Based on the excellent antibacterial effect of hydrogels, the antibacterial mechanism of these hydrogels was proposed (Fig. 4J). Acidic metabolites such as lactic acid produced by bacterial metabolism cause a decrease in the pH of the surrounding environment [45], leading to self-adaptive protonation of the guanidinium and amino groups of CA, causing damage to the integrity of the bacterial cell wall structure. Subsequently, MSCO dissociates to release Cu^2+^ and H_2_O_2_, H_2_O_2_ mediates the Cu^2+^ catalyzed Fenton reaction and the POD-like activity of MoS_2_, respectively, to generate efficient **·**OH for antibacterials. In addition, the PPEL micelles released Lox to catalyze the decomposition of lactic acid to produce H_2_O_2_, and accelerate the production of **·**OH through a positive feedback effect. At the same time, the self-driven cascade NO release triggered by H_2_O_2_ and Cu^2+^ mediated oxidation of GSH and weakened the bacterial resistance to ROS. Moreover, the decrease in the number of bacteria was not caused by the pH change of the bacterial metabolism microenvironment.

### Self-adaptive nanozyme activity

**Figure 5.**
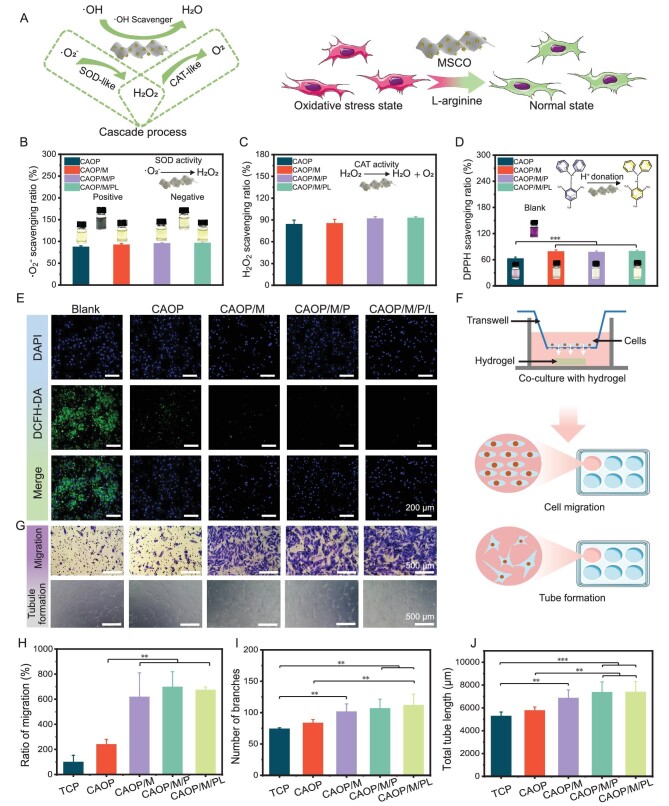
(A) Schematic diagram of hydrogel scavenging ROS and relieving cellular oxidative stress through multiple nanozyme activities. (B) **·**O_2_^−^ scavenging effect through SOD-like activity. (C) H_2_O_2_ scavenging effect through CAT-like activity. (D) DPPH scavenging effect of hydrogels. (E) Fluorescence images of RAW 264.7 cells upon various treatments. (F) Schematic diagram of exploring the effect of different hydrogels treated migration and tube formation of HUVECs. (G) Migration images of HUVECs stained with crystal violet and tube formation images under optical microscope. (H) Migration ratio of HUVECs. (I) Number of branches after different treatments. (J) Total tube length after different treatments. (**P < 0.01, ***P < 0.001).

Considering excess ROS at the wound site during infection causes cellular oxidative stress and delays wound healing, MoS_2_ based nanozyme can convert **·**O_2_^−^ into H_2_O_2_ by superoxide dismutase (SOD)-like activity, and then convert excess H_2_O_2_ into H_2_O and O_2_ by catalase (CAT)-like activity, and convert **·**OH into H_2_O, relieving intracellular oxidative stress under physiological environments (pH 7.4, 37°C) (Fig. 5A) [46,47]. First, the **·**O_2_^−^ scavenging ability of the hydrogel was evaluated *in vitro*. As shown in Fig. 5B, the **·**O_2_^−^ scavenging effect of CAOP was 87.6%, which may be attributed to the guanidine group of CA having a certain degree of ROS scavenging ability [48], while CAOP/M/PL exhibited the best **·**O_2_^−^ scavenging efficiency of 96.6%, which was owing to its SOD-like nanozyme activity.

H_2_O_2_ is the most important for the biologically relevant ROS because it is membrane permeable and has a longer half-life than **·**O_2_^−^ and **·**OH, and thus has the highest intracellular concentration [49]. The CAT-like property of MSCO may accelerate the decomposition of H_2_O_2_ (Fig. 5C). As expected, all hydrogels were able to effectively scavenge more than 84% of H_2_O_2_, and with the addition of MSCO nanozyme, CAOP/M/PL enhanced the scavenging of H_2_O_2_ by exerting its CAT-like activity. In addition, the scavenging activity of the hydrogel on 1,1-diphenyl-2-trinitrophenylhydrazine (DPPH) was evaluated (Fig. 5D). The scavenging effect of CAOP on DPPH was 62.7%, while the scavenging effect on DPPH was significantly increased by the hydrogel containing MSCO nanozyme. The color change of the DPPH solution from dark purple to light yellow also further confirmed the results of the quantitative data (Fig. 5D inset). The **·**OH scavenging effect of the hydrogel under physiological conditions was also evaluated by using terephthalic acid (TA) as a probe (Fig. S28). Similarly, CAOP/M/PL had the most obvious **·**OH scavenging effect.

Based on the proven *in vitro* ROS scavenging properties, H_2_O_2_ induced intracellular oxidative stress were further established to evaluate the cellular level antioxidant effect of the hydrogel. As shown in Fig. 5E, DCFH-DA was used as an intracellular ROS probe, RAW 264.7 cells produced a large amount of ROS (Blank) after being induced by H_2_O_2_, and the intracellular ROS in the hydrogel treatment group was significantly reduced, in which the hydrogel doped with MSCO nanozyme has a more obvious intracellular ROS scavenging ability. Hence, the enhanced ROS scavenging performance of these hydrogels can be attributed to its intrinsic multiple nanozyme properties.

### Biocompatibility and angiogenesis ability

Good biocompatibility is a prerequisite for hydrogels to be used for wound healing. The hemolysis ratios of all hydrogels were below the safe range of 5% (Fig. S29), and the morphology of erythrocytes after co-incubation with hydrogel also confirmed the quantitative results of the hemolysis test (Fig. S30). The biocompatibility of the hydrogel after co-incubation with L929 fibroblasts was evaluated (Fig. S31). These series of hydrogels had good cytocompatibility with cell viability all higher than 90%. Live/dead staining also confirmed these hydrogels have ideal cytocompatibility (Fig. S32).

L-Arginine and Cu^2+^ have been shown to have accelerated angiogenesis [48,50]. The properties of the hydrogel to promote the proliferation of human umbilical vein endothelial cells (HUVECs) *in vitro* were evaluated (Fig. S33). Compared with TCP, HUVECs showed different degrees of proliferation after 1 day of co-incubation with hydrogels, in which the HUVECs proliferation ratio in the CAOP/M/PL group was the most obvious, about 110%. Meanwhile, it was observed that the cell density of all hydrogel groups was significantly higher than that of TCP group by live/dead staining test (Fig. S32). In addition, after 3 days of co-cultivation, compared with the initial TCP group, the proliferation ratios of the TCP, CAOP, CAOP/M, CAOP/M/P and CAOP/M/PL groups reached 148.9%, 151.6%, 153.3%, 153.5% and 155.9%, respectively. Among them, the hydrogel containing MSCO nanozyme has a more obvious effect on promoting the proliferation of HUVECs, which was attributed to the Cu^2+^ released by the slow hydrolysis of CuO_2_ causing cell proliferation. The Cu^2+^ release behavior of the hydrogel in a physiological environment was further verified (Fig. S34). Cu^2+^ released by CAOP/M, CAOP/M/P and CAOP/M/PL reached 2.71, 2.82, and 3.43 μM/L after 48 h, respectively, and the low concentration of Cu^2+^ was helpful in promoting the proliferation of HUVECs [51].

The effect of hydrogels on HUVECs migration and tubule formation was evaluated by Transwell chamber invasion assay (Fig. 5F). Compared with TCP, the number of HUVECs migration after hydrogel treatment was significantly increased (Fig. 5G). The quantitative results of cell migration showed that the cell migration ratio of CAOP/M, CAOP/M/P and CAOP/M/PL was significantly better than that of CAOP (*P* < 0.01) (Fig. 5H). The ability of the hydrogel to induce HUVECs to form tubes was further tested, and the number of branches and tube length was counted. As shown in Fig. 5G, I and J, only a small number of tubules were formed in the TCP group. Hydrogels containing MSCO nanozyme induced HUVECs to form more distinct and interconnected tubule structures, among which CAOP/M/PL had the largest number of tubule branches (about 112) and tubule length (about 7404 μm). In summary, these hydrogels have ideal biocompatibility and good ability to promote angiogenesis and were expected to be candidates for new multifunctional wound dressings.

### Evaluation of infected motion wound healing

**Figure 6.**
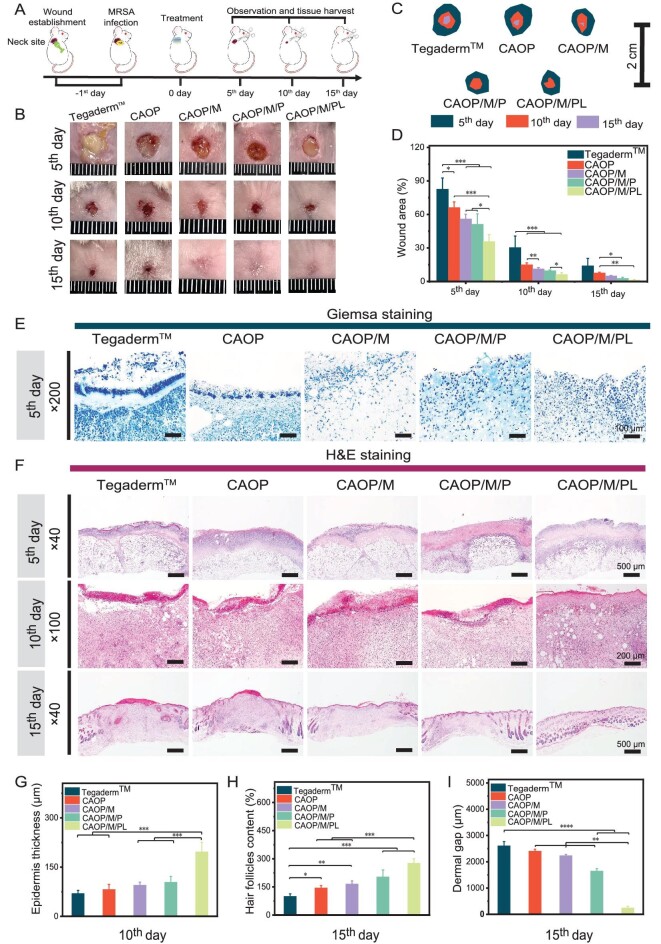
(A) Schematic diagram of the experimental process of *in vivo* infected motion site wound healing. The macroscopic wound closure pictures (B) and wound closure evolution (C) on the 5th, 10th and 15th day. (D) Quantitative analysis of wound areas on the 5th, 10th and 15th day. (E) Giemsa staining of infected tissue on the 5th day. (F) H&E staining of regenerative tissue on the 5th, 10th and 15th day. (G) Epidermis thickness of regenerative tissue on the 10th day. (H) Hair follicles content of regenerative tissue on the 15th day. (I) Dermal gap of regenerative tissue on the 15th day. (*P < 0.05, **P < 0.01, ***P < 0.001, ****P < 0.0001).

Based on the results that the hydrogels demonstrated good mechanical properties, ideal *in vitro* biocompatibility, and excellent *in vitro* antibacterial ability, the ability of these hydrogels to accelerate infected motion wound healing was evaluated by establishing a MRSA infected full-thickness skin defect model of the mouse neck. The progression of the whole *in vivo* study was illustrated in Fig. 6A. The treatments were divided into five groups: Tegaderm™ film, CAOP, CAOP/M, CAOP/M/P and CAOP/M/PL. Wound photographs were taken on the 5th, 10th, and 15th day to assess degree of wound healing. As shown in the visual images (Fig. 6B), evolution of wound closure (Fig. 6C) and quantitative data of wound healing (Fig. 6D), the wounds after different treatments all experienced some shrinkage after the fifth day, while the wounds treated with Tegaderm^TM^ film showed obvious signs of severe infection, and the antibacterial ability of the hydrogel significantly improved the infection of the wound. The wound area of the CAOP group was significantly lower than that of the Tegaderm^TM^ film group (*P* < 0.05). Among all the hydrogel treatment groups, the wound area of the CAOP/M/PL group was about 35.7%, which was significantly better than that of CAOP/M (56%) and CAOP/M/P (51.2%) groups (*P* < 0.05). On the 10th day of wound healing, only 6.25% of the wound area remained in the CAOP/M/PL group with the best therapeutic effect, which was significantly lower than that of the CAOP/M/P group (*P* < 0.05) and the Tegaderm^TM^ film group (*P* < 0.001). On the 15th day of wound healing, the wound surface of the CAOP/M/PL group was almost completely closed and covered the newly generated epidermal structure. Due to the characteristics of difficult wound healing at the motion site, the Tegaderm^TM^ film treatment group still could not effectively achieve wound closure.

The tissue regeneration ability of the composite hydrogel was evaluated by histological analysis. Giemsa staining was used to evaluate the bacterial residue after the fifth day of wound healing (Fig. 6E). The group with wounds treated by Tegaderm^TM^ film showed a large number of aggregated bacteria, indicating the presence of severe infection; the number of bacteria in the CAOP, CAOP/M, CAOP/M/P and CAOP/M/PL groups was significantly reduced, and the bacteria in the CAOP/M/PL treatment group were almost completely eliminated, which was attributed to the self-activating antibacterial ability of the hydrogel induced by the bacterial microenvironment.

The level of inflammatory cells in the regenerated tissue and the status of wound regeneration were evaluated by H&E staining (Fig. 6F). There were many inflammatory cells in the wound after the fifth day of wound healing. Based on the excellent antibacterial ability of the hydrogel, the treatment of the hydrogel reduced the infiltration of inflammatory cells caused by bacterial infection. On the 10th day of wound healing, inflammatory cell infiltration was improved to some extent in all treatment groups, but the Tegaderm^TM^ film group still had the most obvious inflammatory response. The hydrogel treatment group gradually formed the epidermal structure with different degrees. The epidermal thickness formed by the CAOP/M/PL group was more complete than that of other hydrogels (*P* < 0.001) (Fig. 6G), which was attributed to its excellent antibacterial effect during infection treatment, relieving oxidative stress and accelerating angiogenesis after the infection had been eliminated. The wound after the 15th day of healing was observed to have a gradually reduced epidermal gap and improved neovascularization and skin appendages such as hair follicles. Compared with the Tegaderm^TM^ film group, the regenerated hair follicles of CAOP, CAOP/M, CAOP/M/P and CAOP/M/PL groups increased to 144%, 165%, 203% and 276%, respectively (Fig. 6H). In addition, the CAOP/M/PL group also showed the most complete level of dermal regeneration (Fig. 6I). All these results indicated that these hydrogels enhanced wound healing by reducing wound infection and accelerating skin structure reconstruction.

### Immunohistological evaluation of regenerative tissue

The dynamic regulation level of hydrogel on ROS was evaluated by DHE staining (Fig. S35A). Compared with uninfected wounds, the level of ROS in infected wounds increased significantly. The level of ROS in the group with wounds treated by CAOP/M/PL was the highest, which was attributed to the fact that the hydrogel temporarily increased the local ROS content of the wound in response to bacterial infection. After 4 days of treatment, the ROS level of hydrogel treatment groups were significantly reduced, but the Tegaderm^TM^ film group lacked effective anti-infection treatment and still showed a high level of ROS.

Masson staining was used to observe the collagen deposition level after the 10th day of wound regeneration (Fig. S35B). Compared with Tegaderm^TM^ film, the collagen content of the hydrogel group was increased and was more densely arranged; and the CAOP/M/PL group showed the best collagen deposition effect (Fig. S35C).

The levels of inflammation-related factors (TNF-α) and angiogenesis-related factors (VEGF) in the wound were evaluated by immunofluorescence staining (Fig. S35D). After the fifth day of wound healing, the Tegaderm^TM^ film group showed obvious TNF-α expression, and although the CAOP group also had a relatively obvious inflammatory factor signal, it was still significantly lower than that of Tegaderm^TM^ film (Fig. S35E). Among all treatment groups, CAOP/M/PL had the lowest TNF-α expression level, which was attributed to its effective infection control and multiple nanozyme activities to alleviate inflammatory-related factor levels. Angiogenesis-related factors were evaluated after the 10th day of wound healing. It can be seen from Fig. S35D that there was only a small amount of VEGF expression in the Tegaderm^TM^ film group, and the VEGF expression signal in the CAOP group was slightly increased, which was attributed to the angiogenic ability of L-arginine. In addition, the expression level of VEGF in the CAOP/M group was significantly higher than that in the CAOP group (*P* < 0.05) (Fig. S35F), which was due to the Cu^2+^ released from the hydrogel promoting the expression of VEGF, while the expression of VEGF in the CAOP/M/PL group had the best expression level.

## CONCLUSION

In summary, a series of self-activating on-demand antibacterial hydrogel wound dressings triggered by bacterial infection were proposed and fabricated to remodel the regeneration microenvironment, which exhibits smart self-triggered cascade NO release ability activated by bacterial metabolites, and by synergizing the CDT effect enhanced by POD-like activity, the effective treatment of infected motion wounds can be achieved. The hydrogel also exhibited excellent elimination ability against MRSA biofilms and achieved efficient bacterial capture ability. Based on the dynamic interaction ability of Schiff base bonds and phenylboronate bond, the hydrogel exhibited ideal self-healing and mechanical properties, meeting the needs of motion wound protection. In response to the fact that ROS induced cellular oxidative stress after infection elimination, the hydrogel scavenged a variety of ROS by exerting multiple antioxidant activities, and effectively promoted angiogenesis through Cu^2+^ release. In addition, the powerful *in vivo* antibacterial effect, collagen regeneration, and angiogenesis ability of the hydrogel were confirmed by establishing an infected motion wound healing model. Overall, this work highlights the application of bacterial microenvironment-responsive smart hydrogels in the self-adaptive treatment of infected wounds, providing a potential solution for the treatment of related diseases.

## METHODS

The experimental method is obtained in the Supplementary Data.

## Supplementary Material

nwae044_Supplemental_Files
