# Supplementary material for: Bacteria-responsive programmed self-activating antibacterial hydrogel to remodel regeneration microenvironment for infected wound healing
Source: Natl Sci Rev. 2024 Jan 30;11(4):nwae044. doi: 10.1093/nsr/nwae044 (PMC10911815; doi:10.1093/nsr/nwae044)
Supplement: nwae044_Supplemental_Files [file nwae044_supplemental_files.zip › Supplementary data.pdf]

Supporting Information

Bacterial responsive programmed self-activating antibacterial hydrogel to remodel regeneration microenvironment for infected wound healing

Materials

Polyethylene glycol monomethyl ether (mPEG) (Mw: 2000), caprolactone, $Sn(Oct)_2$, hexane-1,6-dioldiacrylate (HDD), 4,4′-trimethylene dipiperidine (TDP), 2,2-diphenyl-1-picrylhydrazyl (DPPH), triethylamine (TEA) and dextran were purchased from Sigma-Aldrich. Lactate oxidase (Lox), polyvinylpyrrolidone (PVP), $MgSO_4$, NaCl, $CuCl_2 \cdot 2H_2O$ and H_2O_2 were purchased from J&K Scientific LTD. MoS_2 nanosheet was purchased from XFNANO Nanjing. Chitosan (Mn =100,000-300,000 Da), L-arginine, 1-ethyl-3-(3-dimethylaminopropyl) carbodiimide hydrochloride (EDC) and N-hydroxysuccinimide (NHS) were purchased from Macklin. Sodium periodate ($NaIO_4$), $KMnO_4$, lactic acid, $Ti(SO_4)_2$, 3,3,5,5-tetramethylbenzidine (TMB), nitrotetrazolium blue chloride (NBT), terephthalic acid (TA), glutathione (GSH) and 5,5'-dithiobis (2-nitrobenzoic acid (DNTB) were purchased from Aladdin. The other reagents were analytically pure and used as received.

Synthesis of triblock copolymer PPE and Lox loaded PPE micelle (PPEL)

Firstly, the synthesis of triblock copolymer PEG-PCL-PAE (PPE) was according to previous report [1]. PEG-PCL was synthesized through ring-opening polymerization of caprolactone using $mPEG_{45}$ (Mw:2000) as the initiator. Briefly, $mPEG_{45}$ (1.0 g, 0.2

mmol) and caprolactone (0.46 g, 4 mmol) were mixed with one drop of $Sn(Oct)_2$ in 50 mL round-bottomed flask. After freeze-degas-thaw cycles for three times, the reaction mixture was stirred at 120 ℃ for 12 h. Then the crude product was dissolved in an appropriate amount of dichloromethane, followed by precipitation into excess diethyl ether. The precipitate was dried under vacuum.

PEG-PCL-PAE was synthesized by a Michael-type addition polymerization of PEG-PCL monoacrylate, HDD and TDP. Firstly, PEG-PCL (1.0 g, 0.21 mmol) and TEA (0.63 mmol) were dissolved in 20 mL of CH_2Cl_2, and a solution of acryloyl chloride (0.63 mmol) in CH_2Cl_2 was added dropwise under argon atmosphere at 0 ℃. The reaction mixture was stirred at room temperature for 24 h. The mixture was washed with saturated sodium carbonate, and then the organic layer was dried over anhydrous $MgSO_4$. The filtrate was precipitated into excess diethyl ether to obtain PEG-PCL monoacrylate. PEG-PCL monoacrylate (0.336 g, 0.07 mmol), HDD (0.21 g, 0.91 mmol) and TDP (0.20 g, 0.97 mmol) were dissolved in 7 mL of $CHCl_3$. After stirred for 72 h at 55 ℃, the solution was precipitated into excess diethyl ether to obtain PEG-PCL-PAE.

The Lox loaded PPE micelle was prepared according to the emulsion/solvent evaporation method. 2 mg of PPE was dissolved in 1 mL of DMSO/ethyl acetate solution (15/85, v/v), followed by addition of 0.4 mL of lactate oxidase (Lox) (1 mg mL^{-1}) solution in water. Then the mixture was sonicated for 5 min to form emulsion. The whole solution was then emulsified into 4 mL of NaCl solution in water (10%, w/v) under sonication for 5 min. This concentrated double emulsion was added into

16 mL of NaCl solution in water (5%, w/v). After evaporation at room temperature for 12 h, the nanoparticle solution was transferred to dialysis bag (MWCO 3500) and dialyzed against deionized water for 48 h. The micelle solution was concentrated ultrafiltration before use.

Synthesis of MSCO nanozyme

First, polyvinylpyrrolidone (PVP) modified CuO_2 nanoparticles were synthesized according to the reported method [2]. Briefly, PVP (0.5 g) was dissolved in an aqueous solution containing $CuCl_2 \cdot 2H_2O$ (5 mL, 0.01 M). Then, NaOH (5 mL, 0.02 M) and H_2O_2 (100 μL) were added sequentially to the above mixture. After stirring for 30 min, the PVP coated CuO_2 nanoparticles were collected by ultrafiltration and washed with water several times. Then, 30 mg of CuO_2 nanoparticles and 30 mg of MoS_2 nanosheets were uniformly mixed and sonicated for 30 min. The mixture was stirred overnight at room temperature, and the CuO_2 coated MoS_2 nanocomposites were collected and washed three times with deionized water. The product was stored at 4 ℃ before use.

Synthesis of L-arginine modified chitosan (CA) and phenylboronic acid modified oxidized dextran (ODP)

L-arginine modified chitosan (CA) was synthesized in 1-(3-dimethylaminopropyl)-3-ethylcarbodiimide hydrochloride (EDC)/N-hydroxysuccinimide (NHS) reaction system. 1 g of chitosan was uniformly dispersed in 100 mL of deionized water, and then 0.5 mL of acetic acid was added to fully dissolve chitosan. At the same time, 2.11 g of L-arginine was dissolved in 1%

acetic acid (25 mL), and then EDC (3.49 g) and NHS (1.15 g) were added to keep the pH of the mixture solution at 5.5 and fully reacted for 2 h. Finally, the activated L-arginine solution was mixed into the chitosan solution, and the pH of the mixed solution was maintained at 5.5 and allowed to fully react overnight. Afterwards, the reaction solution was dialyzed against deionized water for 48 h, followed by lyophilization to obtain the product and stored at 4 ℃.

Sodium periodate ($NaIO_4$) was used to oxidize dextran to obtain oxidized dextran (ODex). 6.34 g of sodium periodate ($NaIO_4$) was dissolved in 50 mL of deionized water and added to 250 mL of a solution containing 10 g of dextran and reacted at room temperature for 6 h. Then, 20 mL of ethylene glycol was added to the mixture and stirred for 2 h to terminate the reaction. Finally, the product was dialyzed in a dialysis bag (MWCO: 3500) for 48 h, and ODex was obtained by lyophilization. Subsequently, 5 g of ODex was dissolved in 100 mL of deionized water, then 0.56 g of 3-aminophenylboronic acid was added. The mixture was stirred at room temperature for 12 h and dialyzed against deionized water for 48 h. The product was lyophilized and stored at 4 ℃.

Preparation of composite hydrogels

Hydrogels were prepared based on dynamic Schiff base reactions and phenylboronate bond. In briefly, 5 wt% CA solution, 5 wt% ODP solution, 10 wt% MSCO nanocomposites dispersion and 20 wt% PPEL micelles dispersion were prepared using phosphate buffered saline (PBS). Then, 150 μL CA, 200 μL ODP, 100 μL MSCO nanocomposites (or 100 μL PBS) and 50 μL PPEL micelles (or 50 μL PBS)

were mixed in 1 mL centrifuge tube and evenly under a vortex shaker. The mixed solution was left to fully react in 37 ℃ shaker, and the obtained hydrogel was collected and stored at 4 ℃.

Characterization of micelle, nanozyme and composite hydrogels

^{1}H nuclear magnetic resonance (^{1}H NMR) (AVANCE III 400 MHz, Bruker) was used to evaluate the chemical structure of CA, Dex, ODex, ODP, PEG-PCL, PEG-PCL-A and PEG-PCL-PAE (PPE). Fourier transform infrared (FT-IR) spectrum (Nicolet 6700, Thermo Scientific, USA) was used to confirm the preparation of CA, ODex, ODP and CAOP hydrogels. Transmission electron microscopy (TEM) (JEOL, JEM-2100) was used to evaluate the morphology of PPE and PPEL micelles, CuO_2 nanoparticles, MoS_2 nanosheets and MSCO nanozyme. The particle size change of PPE micelles loaded with Lox was evaluated by dynamic light scattering (DLS) (Malvern zen3600). The surface charge of PPE and PPEL micelles, CuO_2 nanoparticles, MoS_2 nanosheets, and MSCO nanozyme were evaluated by zeta potential analyzer (Malvern, Zetasizer Nano ZSE).

X-ray photoelectron spectroscopy (XPS) (Thermo Scientific Nexsa) was used to confirm the preparation of MSCO nanozyme and the presence of characteristic chemical bonds. Scanning electron microscopy (SEM) (FESEM; qUTAN FEG 250, FEI) was used to observe the surface morphology and element distribution of MSCO nanozyme, and the pore size change of the hydrogel. Atomic force microscopy (AFM) (Multimode8, Bruker, Germany) was used to evaluate the thickness of the MSCO nanozyme. The crystal structure of MoS_2, CuO_2, MSCO was analyzed by X-ray

diffraction (XRD) (Bruker D8 ADVANCE).

Colorimetric determination of peroxide group

$KMnO_4$ (50 μg mL^{-1}) was dissolved in an aqueous solution containing H_2SO_4 (0.1 M). The mixture was treated with MoS_2 nanosheets, MSCO nanocomposites, or H_2O_2 for 10 min. Subsequently, the UV–vis spectra were measured from 400 to 650 nm.

Swelling and degradation test

A swelling test was used to determine the swelling ratio and in vitro degradation of the hydrogels. Completely gelled wet hydrogels were immersed into PBS (0.01 M, pH 7.4) and then placed at 37 ℃ shaker with a shaking speed of 100 rpm. When reaching the pre-set time interval, the superficial water was removed using a filter paper and the wet hydrogels were weighed. The test was not finished until all the hydrogels were degraded completely. Swelling ratio was calculated using the following equation:

$$\text{Swelling ratio (\%)} = (W_r – W_d)/W_d \times 100\%$$

where W_d and W_r represented the initial weight of the hydrogels and the weight after swelling equilibrium, respectively. The test was repeated four times.

The Weight remaining was calculated using the following equation:

$$\text{Weight remaining (\%)} = W_t/W_i \times 100\%$$

where W_i and W_t represented the initial weight of the wet hydrogels and the wet weight at the pre-set time point, respectively. The test was repeated four times.

Rheological properties

Firstly, 500 μL of hydrogel was placed between 20 mm parallel plates with a gap of

1000 μm and the periphery was sealed by silicone oil to prevent the evaporation of water. Subsequently, time sweep tests were used to evaluate the stiffness of the hydrogels, which were performed at a constant frequency of 1 Hz and a strain of 1% at 25 ℃. The strain amplitude sweep test (γ = 0.1%-1000%) with constant frequency of 10 rad/s at 25°C was performed to detect the hydrogel's critical strain point. The alternate step strain sweep test was performed at a fixed angular frequency (10 rad/s) at 25 ℃. Amplitude oscillatory strains were switched from small strain (γ = 1.0%) to subsequent large strain (γ = 1000%) with 60 s for every strain interval. A shear thinning test with the shear rate varying from 1 1/s to 100 1/s at 25 ℃ was performed to study the effect of shear rate on the hydrogel's viscosity.

Tissue adhesion test

The tissue adhesion strength of the hydrogels was evaluated by using porcine skin via a lap shear test. The skin tissue surfaces were cut into 10 mm × 30 mm rectangle. Then, hydrogel was applied onto the surface of the fresh skin tissue, and another skin was placed on the top of the hydrogel. The contact area of the two skin tissues was kept 10 mm × 10 mm. After that, the samples were placed at 37℃ and keep it sealed for 1 h before the lap shear test. The samples were lap shear tested to failure on an Instron Materials Test system (MTS Criterion 43, MTS Criterion) equipped with a 50 N load cell by using a cross-head speed of 5 mm/min under ambient conditions. All measurements were in septuplicate.

Mechanical performance test.

The mechanical tensile stress−strain evaluation was carried out by the uniaxial tensile

test employing an Instron materials test system (MTS Criterion 43; MTS Criterion) equipped with a 50 N tension sensor at 25 ℃ [3]. All the hydrogel samples were prepared into stripes (30 mm in length × 6 mm in width × 2000 μm in thickness). The tensile strain-stress curves were obtained at a crosshead rate of 5 mm/min.

pH responsive Lox release

After reaching the predetermined time, the samples were taken out for Lox determination. All operations were performed strictly according to the instructions of the kit. The loading of Lox in PPE was also assessed by BCA assay. 500 μL of hydrogel was placed in 4 mL of PBS (pH 5.5, 6.5 and 7.4, respectively) and shaked at 100 rpm at a constant temperature of 37℃. At predetermined time intervals, 1 mL of release solution was removed and then 1 mL of fresh PBS (pH 5.5, 6.5 and 7.4, respectively) was added to maintain a constant volume. The Lox content released from the hydrogel was evaluated by BCA protein concentration assay kit. In short, 20 μL release solution was uniformly mixed with 200 μL BCA working solution, and reacted at 37 ℃ for 30 min, and the Lox release was quantified by measuring the absorbance at 562 nm with a microplate reader. To evaluate the encapsulation efficiency of Lox by PPE micelles, the PPE micelles encapsulated with Lox were placed in PBS at pH 5.5 to completely crack and release Lox. The supernatant was then centrifuged, and the released Lox was assessed by BCA protein assay.

Lox activity analysis

The scavenging capacity of lactic acid (6 mM) by Lox released from the hydrogel in the presence of lipase at different temperature was evaluated by using a commercial

lactic acid test kit (Jiancheng, Nanjing, A019-2-1). All operations were performed strictly according to the instructions of the kit. Hydrogel without loading Lox was used as control. Distilled water was used as blank control. Briefly, 500 μL of hydrogel was placed in PBS containing 6 mM lactic acid (ambient temperature: 25 ℃ and 37°C). After the predetermined time, the hydrogel was removed, and 20 μL supernatant was evenly mixed with 1.2 mL working solution and placed in 37 ℃ water bath for 10 min. Finally, 2 mL of stop solution was added, and the chromogenic solution after the reaction was measured with a microplate reader. The absorbance at 530 nm was used to quantify the residual lactate. Lactic acid content was calculated according to the following formula:

$$\text{Lactic acid content} = \frac{A_t - A_b}{A_n - A_b} \times C_n \times N,$$

Where A_t is the absorbance of the test sample; A_n is the absorbance of the standard; A_b is the absorbance of the blank group; C_n is the concentration of the standard (3 mmol/L); N is the dilution factor of the sample before testing.

Detection of in vitro H_2O_2 production

$Ti(SO_4)_2$ was used as a H_2O_2 indicator whose color changed from colorless to yellow when specifically reacting with H_2O_2. 1 mL of H_2O_2 solution at different concentrations (0.125, 0.25, 0.5, 1 and 2 mM) was added to 1 mL of $Ti(SO_4)_2$ solution (1 mg/mL). Afterwards, the UV-vis spectra of the different mixtures were examined and a plot of absorbance versus H_2O_2 concentration was obtained based on the different absorbance at a wavelength of 410 nm. 500 μL of hydrogel was dispersed in 1 mL of solutions with different pH values (7.4, 6.5, and 5.5, respectively), and the

mixture was stirred at different times (5, 10, 15 and 20 min). Next, the 500 μL supernates were collected by centrifugation and mixed with 1 mL of $Ti(SO_4)_2$ solution (1 mg/mL). After that, the absorbance of different mixtures at 410 nm were examined and the concentrations of H_2O_2 were determined using the standard curve of H_2O_2.

Self-driven NO release performance

To quantify the NO release concentration at each time point, a standard curve was established using commercial $NaNO_2$ (1.25, 2.5, 5, 10, 20 and 40 μM). Under different pH conditions, the samples co-incubated with the hydrogel were taken out after reaching the predetermined time, and reacted with Griess reagent, and the concentration of NO production was quantified by UV-vis spectrophotometer at 540 nm.

Detection of in vitro ·OH production

First, 500 μL of the hydrogel sample was dispersed in 1 mL of PBS with different pH values (7.4, 6.5, and 5.5, respectively), and the mixture was stirred at different times (5, 10, 15, and 20 min). Next, the 500 μL supernates were collected by centrifugation and mixed with 1 mL of TMB solution (40 μg mL^{-1}). After that, the absorbance of different mixtures at 650 nm was examined.

Detection of in vitro GSH elimination

Briefly, 500 μL of hydrogel was dispersed in 1 mL of solutions with different pH values (7.4, 6.5, and 5.5, respectively), and the mixture was stirred at different times (5, 10, 15, and 20 min). Next, the 500 μL supernates were collected by centrifugation and mixed with 1 mL DTNB (10 mM) solution containing 10 mM GSH. After that,

the absorbance of different mixtures at 410 nm were examined. In addition, GSH solutions with different pH were used as controls.

Self-activating antibacterial ability

The self-activating antibacterial activity of hydrogel for *E. coli* and MRSA under different pH was performed. In brief, 200 μL of hydrogels was prepared into a 48-well microplate (Costar). After completely gelled, 10 μL of bacterial suspension (10^7 CFU mL^{-1}) in sterilized PBS (pH 7.4, 6.5 and 5.5, respectively) was added onto each hydrogel surface in 48-well culture plate. The inoculated hydrogels were incubated for 2 h at 37 ℃ and the relative humidity of the inside of microplate was not less than 90%. At the end of that time, 1 mL of sterilized PBS was then added to each well to re-suspend any bacterial survivor. 10 μL of bacterial suspension (10^7 CFU mL^{-1}) suspended in 1 mL of PBS was used as a negative control. After incubated for 18 h at 37 ℃, the colony-forming units on the agar plate (Petri dish) were counted. Tests were repeated four times for each group.

Bacterial morphology was examined by SEM after the antibacterial experiment. After co-incubating the hydrogel with bacteria for 2 h, hydrogels were immobilized with 2.5% (v/v) glutaraldehyde for 2 h and then sequentially dehydrated in a graded ethanol series for 10 min (30%, 50%, 70%, 90%, 100%). Finally, the bacterial morphology and integrity were examined by SEM after drying and coating with gold.

After the antibacterial experiment, MRSA were stained with the help of live/dead staining kit to further evaluate the antibacterial ability of the hydrogels. The bacterial suspension (10^7 CFU mL^{-1}) was incubated on hydrogels for 2 h under different pH,

the remaining bacteria were then re-suspended using sterile PBS and stained with a live/dead staining kit for 30 min, and the bacteria were observed by inverted fluorescence microscopy (IX53, Olympus). After staining, live bacteria which harbored an intact membrane presented green fluorescence while dead bacteria which harbored an impaired membrane structure principally exhibited red fluorescence.

In vitro MRSA biofilm elimination test

Briefly, 500 μL of MRSA (1×10^8 CFU mL^{-1}) in MHB (Mueller–Hinton broth) medium was added into 48-well plates. Then, bacteria were cultured at 37 ℃ for 48 h, and media were freshly replaced every 24 h. Finally, the medium was removed, and the unattached bacteria were gently washed away with sterile PBS for three times, and the resulting in biofilm on 48-well plates was harvested.

200 μL of hydrogel was added to the bacterial biofilm containing PBS (pH 6.5) and then incubated at 37℃ for 6 h without shaking. The group without hydrogel was used as a blank control. After incubation, the hydrogel was removed and rinsed with PBS three times to remove floating bacteria. Then, 500 μL of anhydrous alcohol was added to fix the biofilm and it was incubated at 4℃ for 15 min. Subsequently, remove the alcohol, 300 μL of 1% crystal violet dye was used to stain the biofilm for 30 min. After staining, the biofilm was washed 3 times with PBS and dried at room temperature. Finally, 500 μL of anhydrous alcohol was added to each well and incubated for 30 min, and the absorbance of the solution was read at 550 nm with a microplate reader (Molecular Devices). Tests were repeated four times for each group.

Bacteria capture capability

The monocolony of MRSA and *E. coli* on solid Luria-Bertani (LB) agar plates were transferred to 14 mL of liquid LB culture medium and incubated in shaking table (180 rpm) at 37 ℃ for 12 h. Then the bacteria solution was diluted 3 times by PBS buffer with different pH for the bacteria capture experiment. The 500 μL cylindrical hydrogels were put into the test tube, and 1.5 mL of bacterial solution was added, then the test tube was placed at room temperature for 1 h to trap bacteria. After that, removing the hydrogels, the bacterial concentration was detected by measuring the optical density at 600 nm (OD 600 nm).

Nanozyme activity

SOD-like activity of hydrogels was determined by calculating the inhibition ratio of the photoreduction of nitrotetrazolium blue chloride (NBT). In a typical experiment, riboflavin (20 μM), methionine (12.5 mM), NBT (75 μM), and 500 μL samples were mixed in 2 mL PBS (pH 7.4) and treated with a constant light intensity for 30 min at 25 ℃. Two-milliliter of the supernatant was collected, the full-scan curve was measured, and absorbance values at 560 nm were obtained. The inhibition ratio was calculated using equation:

$$\cdot O_2^- \text{ scavenging ratio (\%)} = \frac{A_o - A_n}{A_p - A_n} \times 100\%,$$

where A_o, A_n, and A_p represent the absorbance values of the sample, negative control, and positive control, respectively.

CAT-like activity of hydrogels was determined by $Ti(SO_4)_2$ colorimetric method. 3 mL of H_2O_2 (1 mM, diluted with PBS) and 500 μL of samples were incubated in oven at 37 ℃ for 4 h. Then 1 mL of supernatant was taken out and 2 mL of $Ti(SO_4)_2$ (1 mg

mL^{-1}) solution was added for 0.5 h. Then UV–vis spectrophotometer was used to scan the absorbance curve of 300–600 nm wavelength range.

The ·OH scavenging efficiency of hydrogels was characterized by measuring the fluorescence of 2-hydroxyterephthalic acid. As a no fluorescent compound, terephthalic acid (TA) could capture ·OH to generate 2-hydroxyterephthalic acid. 500 μL of hydrogel was put into 2 mL PBS containing TA (0.5 mM) and H_2O_2 (1 mM), and incubated for 12 h at 37 ℃. Finally, fluorescence analysis of the system was performed.

Intracellular ROS scavenging

Briefly, RAW 264.7 cells in a 48-well plate (3×10^4 cells/well) were incubated with hydrogels for 24 h. Next, 500 μM H_2O_2 was added to the culture media to induce oxidative stress for 1 h. Then, the media and samples were aspirated, and the cells were washed carefully with PBS for 2 times. The cells were incubated with 10 μM DCFH-DA solution and DAPI at 37 ℃ for 1 h in dark. The fluorescence images were observed by confocal microscope.

Hemostatic ability

The hemostatic effect of the hydrogel was evaluated by mice liver trauma model and liver incision model. As for mice liver trauma model, the mice (Kunming mice, weighing ~35 g, female) were anesthetized by injecting 10 wt% chloral hydrate (0.3 mL per 100 g weight). A pre-weighted filter paper on a paraffin film was placed beneath the liver, bleeding from the liver was induced using a 20 G needle with the corkboard tilted at about 30° and 250 μL of the hydrogel or gauze (1 cm × 1 cm) was

immediately applied on the bleeding site by using the syringe. The weight of the filter paper with absorbed blood was measured until stop bleeding and compared with a control group (no treatment after pricking the liver). As for mice liver incision model, the mice (Kunming mice, weighing ~35 g, female) were anesthetized by injecting 10 wt% chloral hydrate (0.3 mL per 100 g weight). A pre-weighed filter paper on a paraffin film was placed beneath the liver. Then, bleeding from the liver was induced by creating a wound (4 mm long, 2 mm deep) by using a scalpel and 250 μL of the hydrogel or gauze (1 cm × 1 cm) was immediately applied to the bleeding site using the syringe. The weight of the filter paper with absorbed blood was measured until stop bleeding and compared with a control group (no treatment after pricking the liver). All the measurements were carried out with 5 times of repetition.

For rat liver volume defect model, the SD rats (weight of 180−220 g, female) were fixed on the surgical cork-board and then 10% chloral hydrate was injected into rats enterocoelia to anesthetize them (0.3 mL per 100 g weight of animal). Following that, the rat experienced an abdominal incision to expose its liver, the serous fluid around the liver was carefully removed, and then a columniform liver volume defect (with a diameter of 5 mm and height of 4 mm) was made in liver using biopsy needle (inner diameter of 5 mm) and surgical scissors. Immediately after wiping off the blood by using gauze, the hydrogel was injected onto the defect hole or gauze was placed onto the defect hole. During the hemostatic process, the weighed gauze was used to absorb the flowing blood. The hemostatic time and blood loss were recorded accordingly. Each group contained 7 rats.

Biocompatibility test

Biocompatibility of the hydrogels was evaluated by hemolysis activity and L929 fibroblast biocompatibility. As for hemolysis activity test, erythrocytes were separated by centrifugation (at 1,000 rpm) from the mice blood for 10 min. The obtained erythrocytes were washed for three times with PBS buffer and then diluted to a final concentration of 5% (v/v) by PBS. Hydrogel samples (500 μL) mixed with erythrocytes stock (500 μL) was added to a 24-well microplate, then shaken in an incubator at 37 ℃ for 1 h with a shaking speed of 100 rpm. 0.1% of Triton X-100 was used as the positive control while PBS buffer was used as the negative control. After that, the microplate well contents were centrifuged (at 1,000 rpm) for 10 min and the supernatant (100 μL) was then introduced into a new 96-well microplate. The absorbance of the solution was recorded at 540 nm by a microplate reader (Molecular Devices). The hemolysis percentage was calculated from the formula:

$$\text{Hemolysis (\%)} = [(A_p - A_b) / (A_t - A_b)] \times 100\%,$$

where A_p was the absorbance value for erythrocytes treated with samples. A_t was the absorbance value for the 0.1% Triton X-100 positive control and A_b was the absorbance value for PBS negative control. In addition, the morphology of erythrocytes after co-incubation with the hydrogel was observed by optical microscopy.

The cytocompatibility and cell proliferation treated with hydrogel was evaluated by alamarBlue® assay and LIVE/DEAD® Viability/Cytotoxicity Kit assay, respectively. First, L929 cells were seeded in 48-well plate at a density of 10000 cells/well. After

cultured for 24 h, the hydrogel disks were introduced into the wells. After being co-incubated for 1, 2 and 3 days, the hydrogel disks and medium were removed and 20 μL of alamarBlue® reagent in 200 μL complete growth medium was then added into each well. The plate was incubated for 4 h in a humidified incubator containing 5% CO_2 at 37°C. After that, 100 μL of the medium in each well was transferred into a 96-well black plate (Costar). Fluorescence was recorded using 560 nm as the excitation wavelength and 600 nm as the emission wavelength by using a microplate reader (Molecular Devices) according to the manufacturer's instructions. Cells seeded on plate without hydrogel disks served as the TCP group. Tests were repeated four times for each group. Cell adhesion and viability were observed under an inverted fluorescence microscope (IX53, Olympus).

Angiogenic capacity

The hydrogel was co-incubated with HUVECs cells to evaluate the ability to promote HUVECs proliferation. HUVECs cells were seeded in 48-well plate at a density of 6000 cells/well. After cultured for 12 h, the hydrogel disks were introduced into the wells. After being co-incubated for 1, 2 and 3 days, the hydrogel disks and medium were removed and 20 μL of alamarBlue® reagent in 200 μL complete growth medium was then added into each well. The plate was incubated for 4 h in a humidified incubator containing 5% CO_2 at 37°C. After that, 100 μL of the medium in each well was transferred into a 96-well black plate (Costar). Fluorescence was recorded using 560 nm as the excitation wavelength and 600 nm as the emission wavelength by using a microplate reader (Molecular Devices) according to the manufacturer's instructions.

Cells seeded on plate without hydrogel disks served as the TCP group. Tests were repeated four times for each group. Cell adhesion and viability were observed under an inverted fluorescence microscope (IX53, Olympus).

As for HUVECs migration test, Matrigel (BD Biosciences, USA), which had been placed at 4 ℃ overnight, was used to treat the Transwell chamber. Medium containing 10% Foetal Bovine Serum (FBS) was added to the bottom chamber containing the hydrogels, while HUVECs was suspended in serum-free medium at a density of 5.0×10^4 cells/mL were seeded in the top chamber, respectively. After 24 h of indirect co-culture, the top chamber was removed, and the upper side of the membrane was wiped with wet cotton swabs. The cells on bottom of chambers were then fixed with 4% paraformaldehyde for 20 min and stained with crystal violet staining solution for 30 min (Beyotime). The chamber containing cells was placed on a glass slide and photographed using an optical microscope (Olympus).

Tubule formation assay of HUVECs was used to measure the ability of vascularization. Matrigel (BD Biosciences, USA) without growth factors was first thawed overnight at 4 ℃. The 48-well culture plate with 100 μL Matrigel spread on its bottom was incubated for 30 min at 37 ℃ to gelatinize. Thereafter, HUVECs at a density of 5×10^4 cells/well were seeded on culture plate containing Matrigel. After co-culturing the hydrogels with cells in a cell incubator for 12 h, the cells on culture plate were washed with PBS and the tubule network of HUVECs was imaged with an optical microscope (Olympus).

In vivo subcutaneous anti-infection experiment

Kunming mice (female, ~35 g) were used in the animal study. All the animal experiments were approved by the institutional review board of Xi'an Jiaotong University. To evaluate the in vivo antibacterial efficacy of the hydrogels, four subcutaneous abscesses were experimentally created in each test mouse. Briefly, the mice were firstly anesthetized using 10% chloral hydrate. After shaving and disinfection, a subcutaneous injection of MRSA (10^8 CFU/mL, 10 μL) was given on the shaved back of the test animals. Five mice were used in this study. At 24 h following the injection of bacteria, infected abscesses had formed subcutaneously in each test mouse. The hydrogels were then directly applied onto the infected wound. Each test mouse received five MRSA infections, and these were then treated under one of the following five experimental conditions: PBS; CAOP; CAOP/M; CAOP/M/P and CAOP/M/PL. Following treatments, the mice were sacrificed, and the infected tissues were harvested and analyzed by standard plate count methods.

In vivo infected motion wound healing evaluation

All the animal experiments were approved by the institutional review board of Xi'an Jiaotong University. Kunming mice (female, 35-40 g) were used for the test. Before the surgery, the mice were acclimatized for 1 week. The mice were anesthetized by intraperitoneal injection of 10% chloral hydrate, and then the dorsal region of mouse above the back but below the head was shaved for further surgery. One full thickness wounds with diameter of ~8 mm was made on neck site of the mouse midline, and 10 μL MRSA (10^8 CFU/mL) was injected into the wound to establish infection. First group was dressed by Transparent Film Dressing (3M Health Care, USA). Second

group was dressed by Transparent Film Dressing and CAOP, and the third group was dressed by CAOP/M and Transparent Film Dressing. The fourth group was dressed by Transparent Film Dressing and CAOP/M/P, and the fifth group was dressed by Transparent Film Dressing and CAOP/M/PL. Each group contains 15 mice. For wound area monitoring, on the 5th, 10th, and 15th day, the wound area was photographed. The wound area was calculated by Image J software. Wound area (%) was calculated using the following equation:

$$\text{Wound area} = (\text{area (n day)})/ (\text{area (0 day)}) \times 100\%.$$

Histology and immunohistochemistry analysis

To evaluate the epidermal regeneration and inflammation in wound area, the collected skin samples were fixed with 4% paraformaldehyde for 1 h, embedded in paraffin, and then cross sectioned to 4 μm thickness slices. The obtained slices were then stained by Haematoxylin-Eosin (Beyotime, China), Dihydroethidium (DHE) Masson’s trichrome stain. All slices were analyzed and photographed by microscope (IX53, Olympus, Japan). The regenerated skins from the wound site were also excised on the 5th, 10th, and 15th day for immunofluorescence staining. The fixed and frozen sections were stained with TNF-α (Affinity Biosciences) and VEGF (Affinity Biosciences), respectively. FITC-conjugated goat anti-mouse IgG (cwbiotech) and FITC-conjugated goat anti-rabbit IgG (cwbiotech) were used as the secondary antibody to reveal TNF-α expression and VEGF expression. The nuclei were stained with DAPI containing mounting solution. Slides were observed under an inverted fluorescence microscope (IX53, Olympus).

Statistical analysis

The above experiments were repeated at least three times. GraphPad Prism 8.0 was applied to calculate significant differences based on a one-way analysis using Student's t-test. Values of *$p < 0.05$, **$p < 0.01$, ***$p < 0.001$ and ****$p < 0.0001$ were considered statistically significant, respectively.

RESULTS

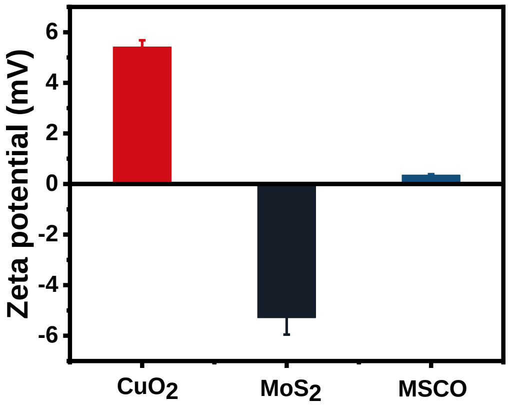

Figure S1. Zeta potential of CuO_2 nanoparticle, MoS_2 nanosheet and MSCO nanocomposite.

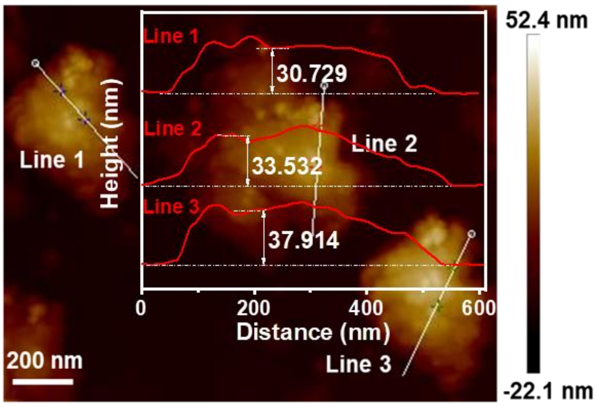

Figure S2. AFM image and thickness distribution of MSCO nanocomposite.

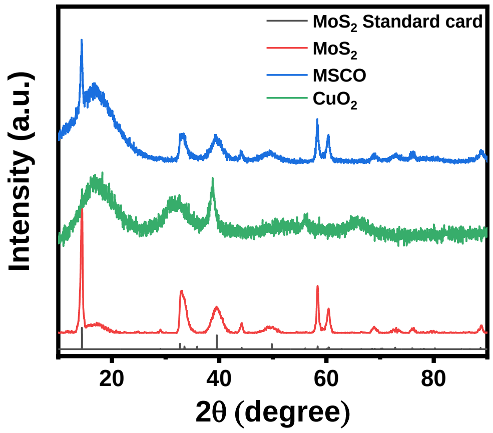

Figure S3. XRD pattern of MoS_2, CuO_2 and MSCO.

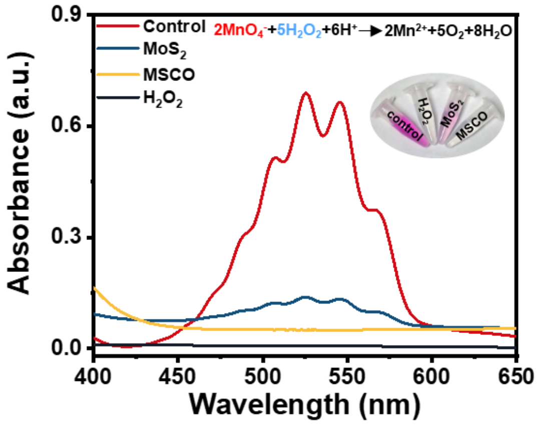

Figure S4. Colorimetric analysis demonstrating the presence of peroxy groups in MSCO nanocomposite.

The color of MnO_4^- in acidic solution disappears after treatment with MSCO nanocomposite, which was attributed to the reduction of pink MnO_4^- to colorless Mn^{2+} by peroxy groups.

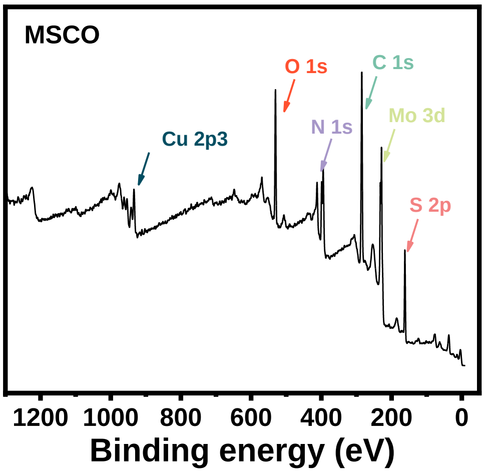

Figure S5. XPS spectra of MSCO nanocomposite.

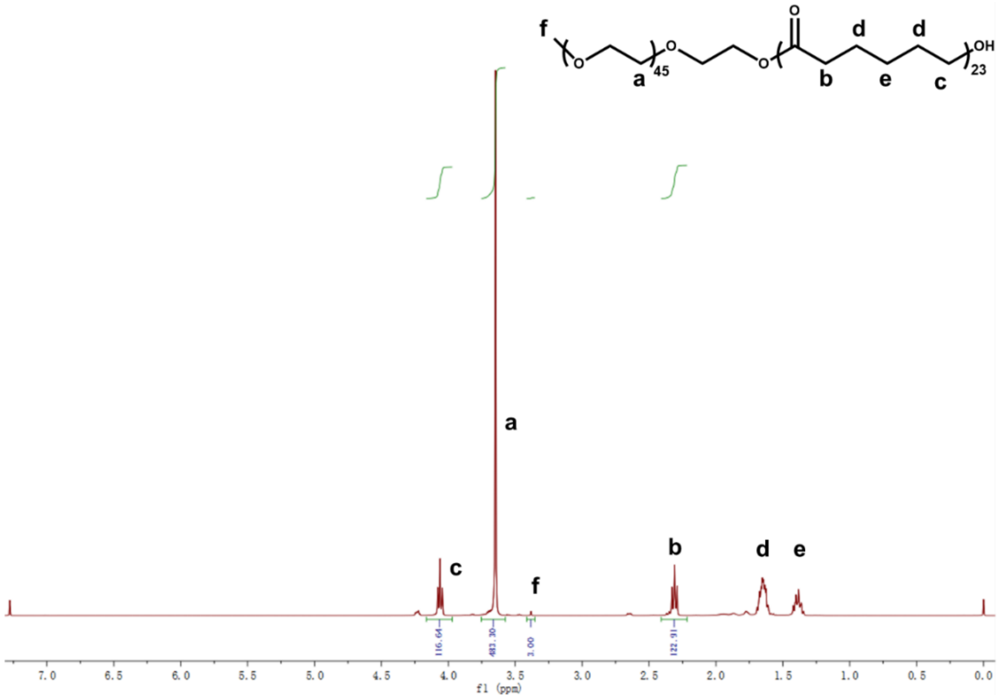

Figure S6. ^1H NMR spectra of PEG-PCL.

The degree of polymerization (DP) of caprolactone was estimated to be 23 by

calculating the peak integration ratio of $-OCH_2CH_2-$ protons of PEG at 3.64 ppm and $-OCOCH_2-$ protons of PCL at 2.3 ppm.

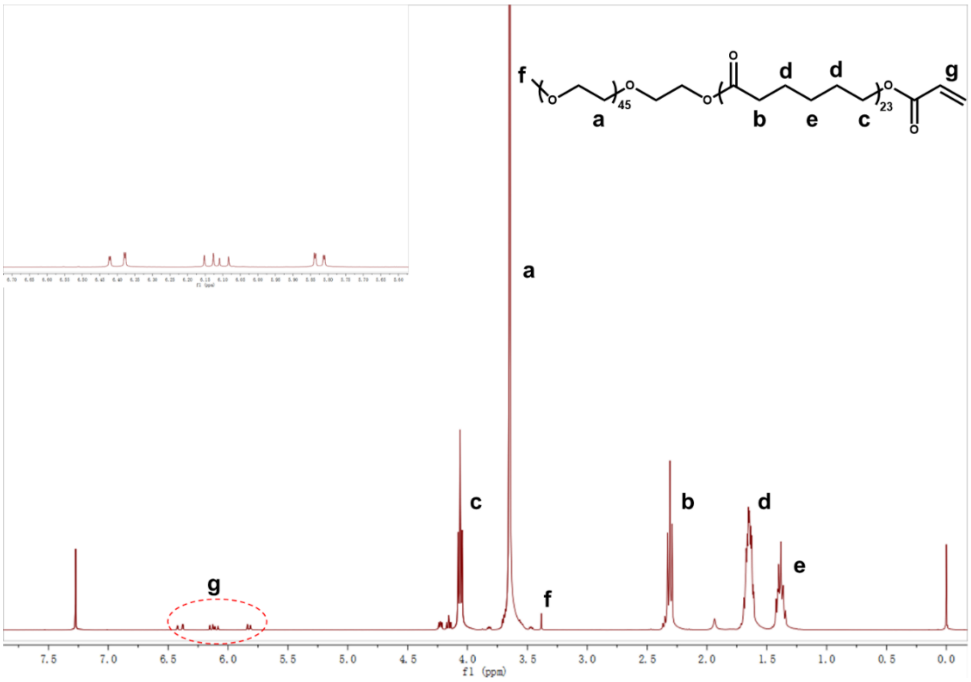

Figure S7. ^{1}H NMR spectra of PEG-PCL-A.

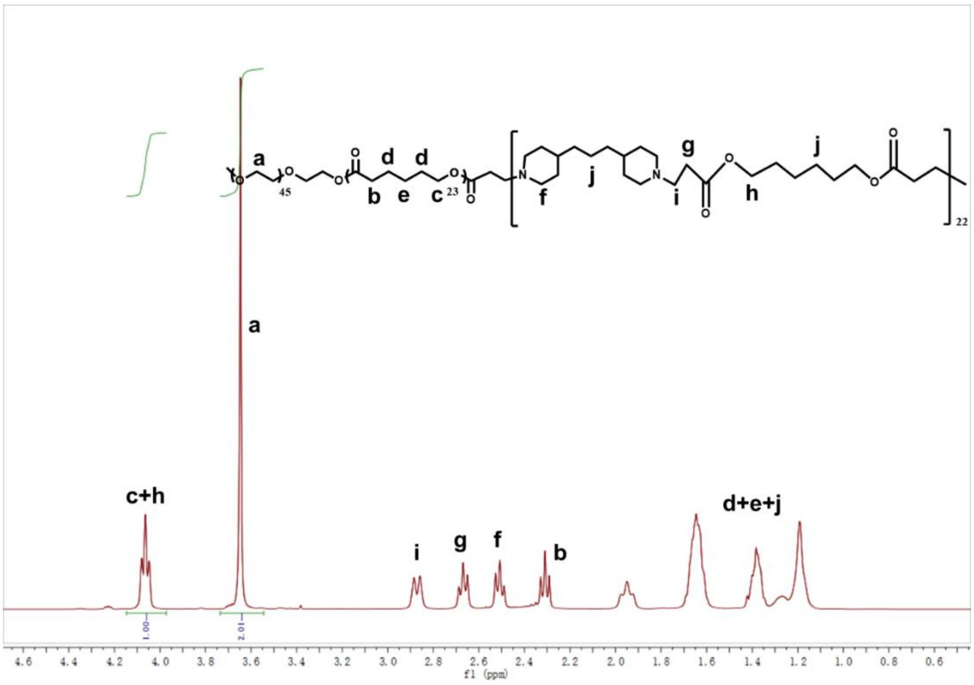

Figure S8. ^{1}H NMR spectra of PEG-PCL-PAE.

The DP of polymerization of PAE was determined to be 22 by calculating the peak

integration ratio of $-OCH_2CH_2-$ protons of PEG at 3.64 ppm and $-OCH_2CH_2-$ protons of PCL at 4.06 ppm.

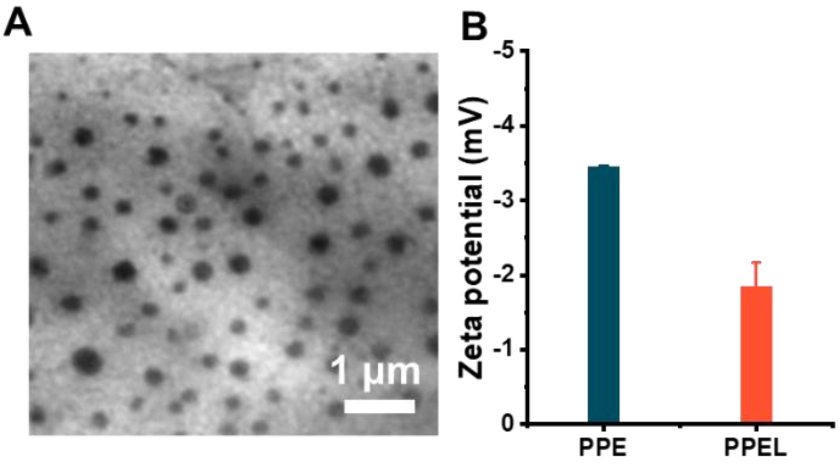

Figure S9. (A) TEM image of PPEL micelle; (B) Zeta potential of PPE and PPEL micelle.

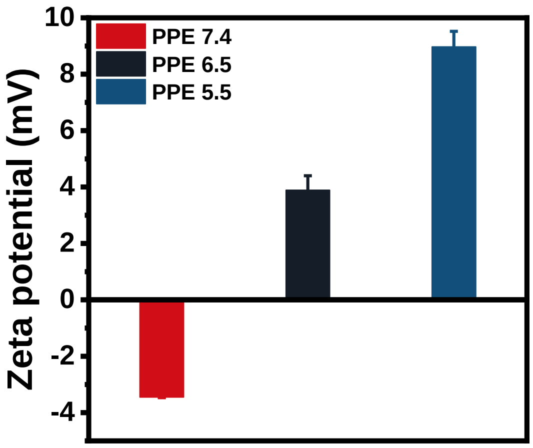

Figure S10. Zeta potential of PPE under pH 7.4, 6.5 and 5.5.

Under physiological conditions (pH 7.4), PAE was negatively charged (−3.45 mV) due to its deprotonation, while the zeta potentials of PAE after re-protonation were 3.89 mV and 8.97 mV at pH 6.5 and 5.5, respectively.

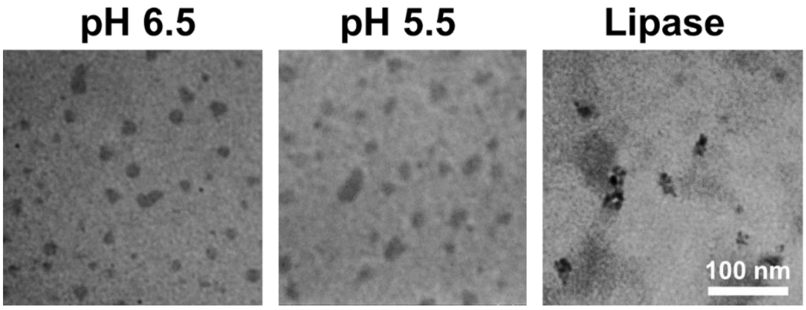

Figure S11. TEM images of PPE micelle under pH 6.5, 5.5 and presence of lipase.

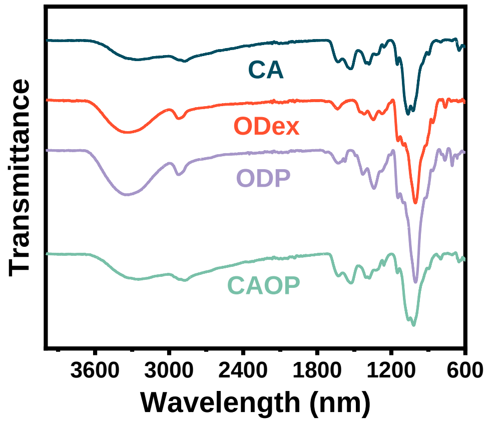

Figure S12. FT-IR spectra of CA, ODex, ODP and CAOP hydrogel.

For the FT-IR spectra of CA, the characteristic peaks of L-arginine at 1630 and 1410 cm^{-1} (guanidine group and COO- symmetric bending, respectively), indicated the effective conjugation of L-arginine on chitosan polymer chains. The characteristic peak at 1730 cm^{-1} was attributed to the stretching vibration of the aldehyde group (C=O), which indicated that dextran was successfully oxidized. Compared with ODex, a new peak appearing at 1575 cm^{-1} in the ODP spectrum was assigned to the imine bond (C=N), which was attributed to the Schiff base bond formed between the aldehyde group of ODex and 3-aminophenylboronic acid. The newly formed peaks at 1340 cm^{-1} were attributed to the B-O tensile vibration. In addition, ODP still had a peak at 1730 cm^{-1}, which indicated that ODP retained its aldehyde group after

modification with 3-aminophenylboronic acid. In the FT-IR spectrum of CAOP hydrogel based on CA and ODP, the characteristic peak of aldehyde groups at 1730 cm^{-1} disappeared, which indicated that the hydrogel was prepared by Schiff base reaction.

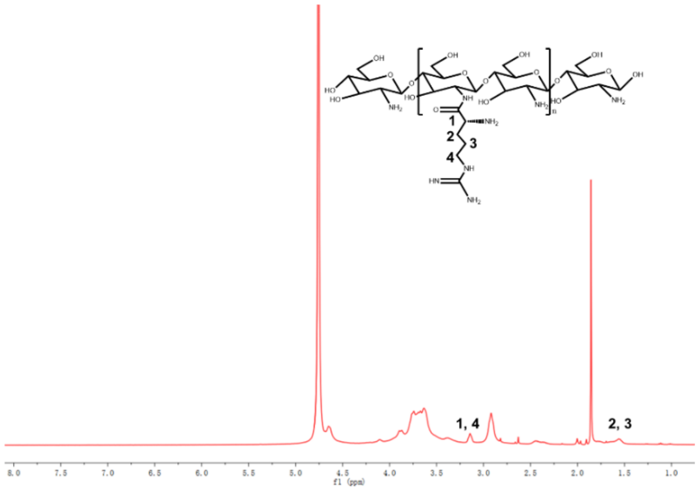

Figure S13. ^{1}H NMR spectra of CA.

In the ^{1}H NMR spectra of CA, the characteristic peaks located at 3.75 and 1.75 ppm confirmed the successful preparation of CA.

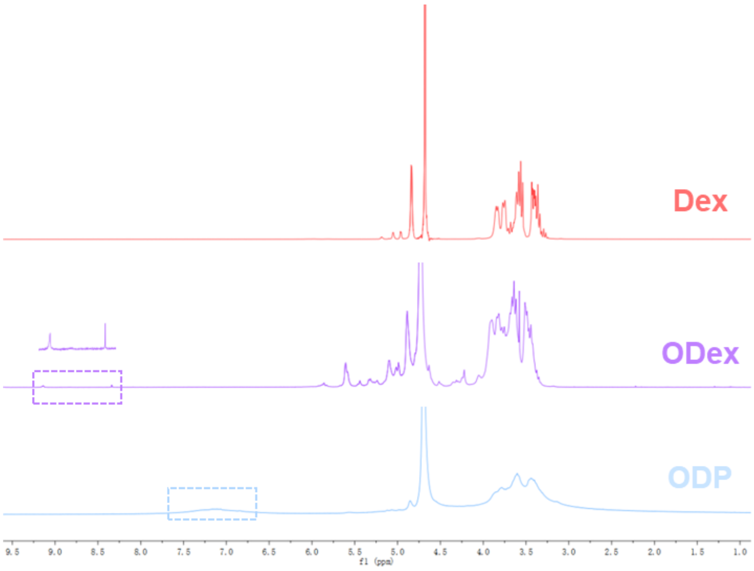

Figure S14. ^{1}H NMR spectra of Dex, ODex and ODP.

The presence of phenyl group protons from 6.7 to 7.7 ppm in ODP verified the

successful grafting of 3-aminobenzeneboronic acid.

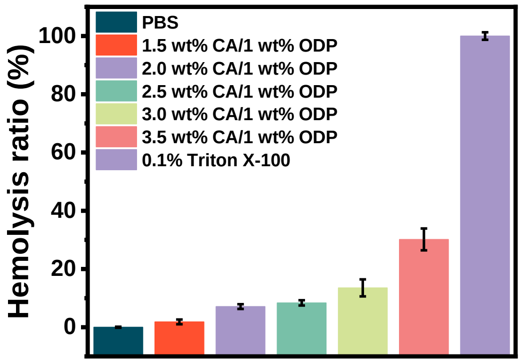

Figure S15. Hemolysis ratio of hydrogels with different CA concentration.

When the CA concentration reached 2.0 wt%, the hemolysis ratio of the hydrogel reached 7.11%, while the hemolysis ratio of 1.5 wt% CA was within the safe range (1.84%).

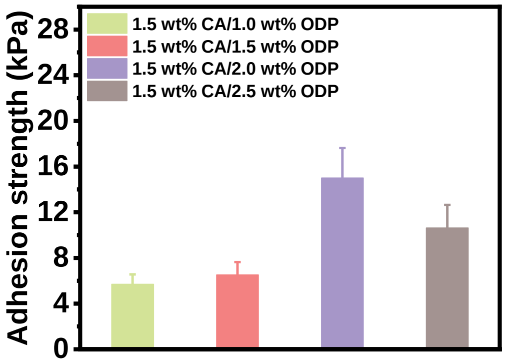

Figure S16. Adhesion strength of hydrogels with different ODP concentration.

The effect of different ODP concentrations on the adhesion strength of hydrogels containing 1.5 wt% CA was further explored. When the ODP concentration was 2.0 wt%, the hydrogel showed the best adhesion strength.

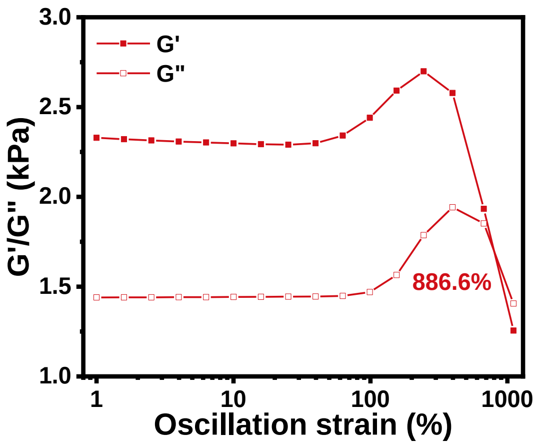

Figure S17. Strain amplitude sweep test of CAOP/M/PL.

The critical strain point required for the collapse of the hydrogel network structure was evaluated before the self-healing test. When the oscillatory strain applied to the hydrogel was greater than 886.6%, the storage modulus of the hydrogel was lower than the loss modulus, indicating that the structure of the hydrogel was destroyed.

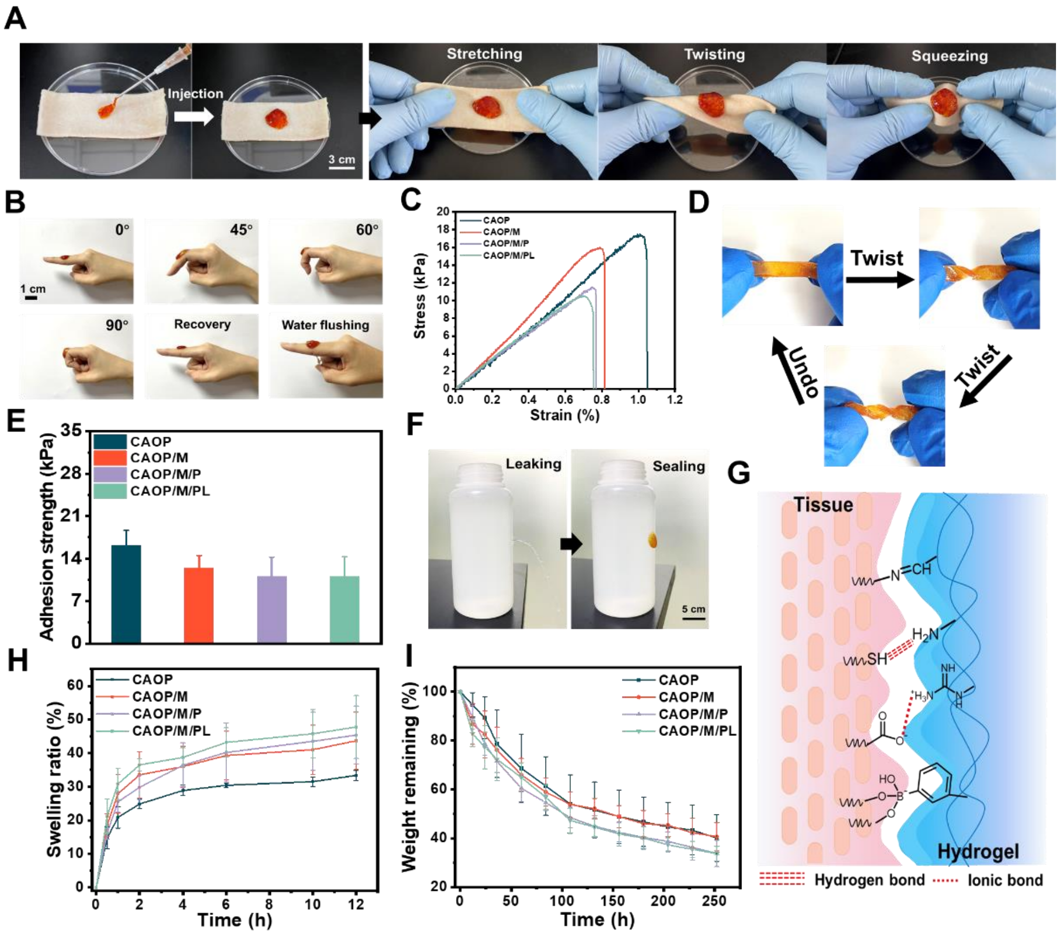

Figure S18. (A) Injectable demonstration and adhesion robustness of hydrogel. (B) Presentation of hydrogel adhered to finger joints under different motion angles. (C) The tensile stress-strain curves of hydrogels. (D) Presentation of the twisted hydrogel. (E) Adhesion strength of hydrogels. (F) Presentation of hydrogel preventing liquid leakage. (G) Schematic diagram of the adhesion mechanism of the hydrogel. Swelling (H) and degradation (I) properties of hydrogels.

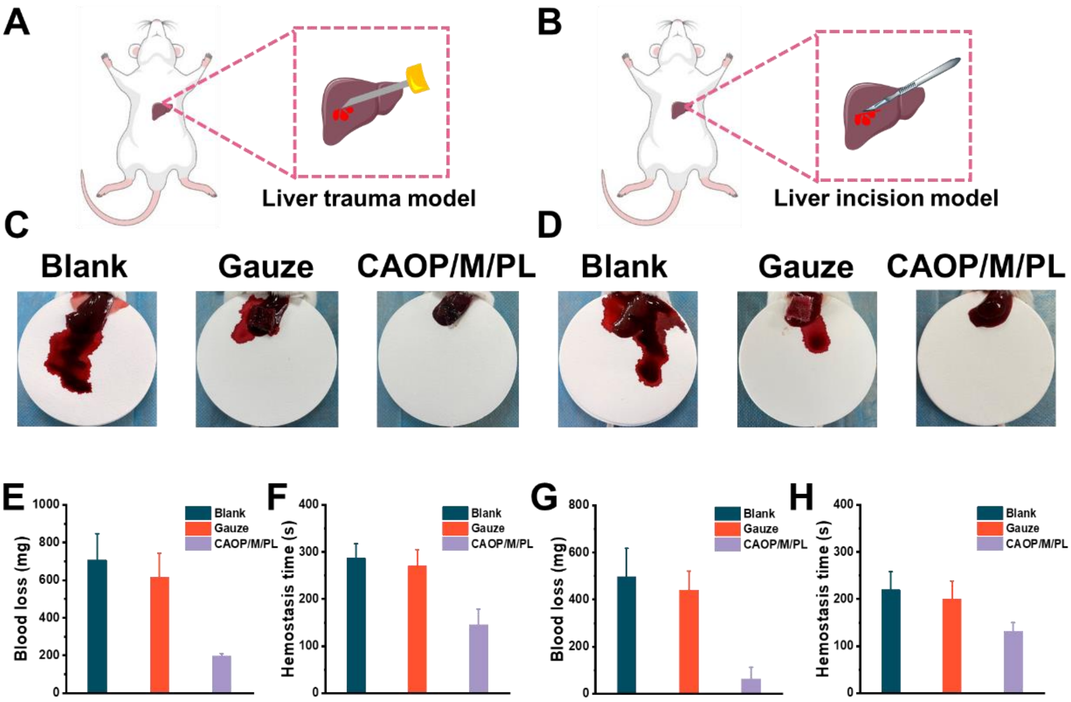

Figure S19. Schematic illustration of (A) mouse liver trauma model and (B) mouse liver incision model; Presentation of the hemostatic process in a (C) mouse liver trauma model and (D) mouse liver incision model; Blood loss of (E) mouse liver trauma model and (G) mouse liver incision model; Hemostasis time of (F) mouse liver trauma model and (H) mouse liver incision model.

Based on the good tissue adhesion exhibited by the hydrogel, the hemostatic effect of CAOP/M/PL in vivo was evaluated through a mouse liver trauma model (Figure S19A) and mouse liver incision model (Figure S19B). Gauze was used as a commercial hemostatic agent as a control group. Compared with the blank group and gauze group, the hydrogel was able to seal the wound after bleeding and maintain good tissue adhesion, and no significant blood loss was observed (Figure S19C, D). For the mouse liver trauma model, the hydrogel was able to reduce the blood loss to

198 mg compared with the blood loss of the blank group (707 mg) and the gauze group (615 mg) (Figure S19E). In addition, the hemostasis time of hydrogel was reduced to 146 s compared with the gauze group (271 s) (Figure S19F). For the mouse liver incision model, the blood loss in the blank group, gauze group and hydrogel group were 496 mg, 441 mg and 64 mg, respectively (Figure S19G). Compared with the blank group, the hemostasis time of the hydrogel group was reduced from 220 s to 131 s (Figure S19H).

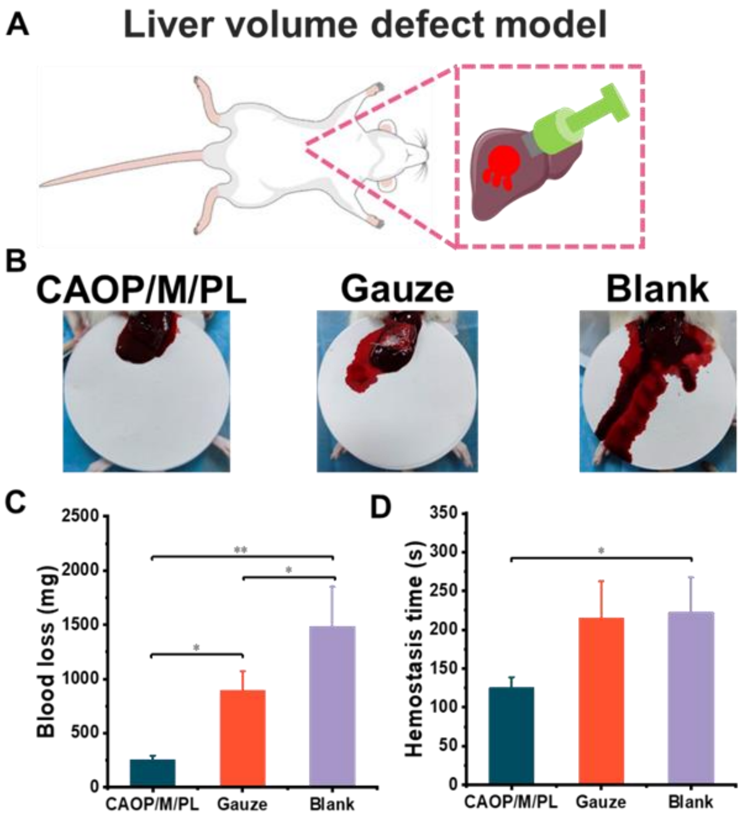

Figure S20. (A) Schematic illustration of rat liver volume defect model; (B) Presentation of the hemostatic process; (C) Blood loss of rat liver volume defect model; (D) Hemostasis time of rat liver volume defect model. (*p < 0.05, **p < 0.01)

Rat liver volume defect model was further established to evaluate the hemostatic

effect of the hydrogel on hemorrhage from visceral volume defects (Figure S20A). The hydrogel sealed the bleeding wound through good tissue adhesion and showed only a small amount of bleeding, while the gauze group and the blank group showed a large amount of blood loss due to the lack of effective occlusion and procoagulant components (Figure S20B). Compared with the blood loss of blank group of about 1486 mg and gauze group about 893 mg (Figure S20C), the blood loss in the hydrogel group was significantly reduced to about 253 mg ($p < 0.05$). In addition, the hemostasis time of the hydrogel group was reduced from 223 s to 125 s compared with the blank group ($p < 0.05$) (Figure S20D). In conclusion, CAOP/M/PL hydrogel has more effective bleeding control than commercial gauzes, and it is expected to become a new type of hemostatic agent for wound hemostasis.

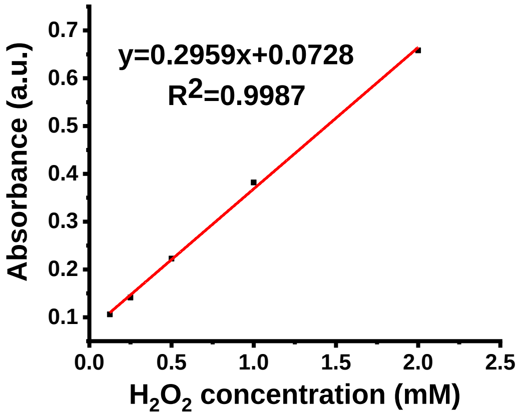

Figure S21. Standard curve of H_2O_2 concentration.

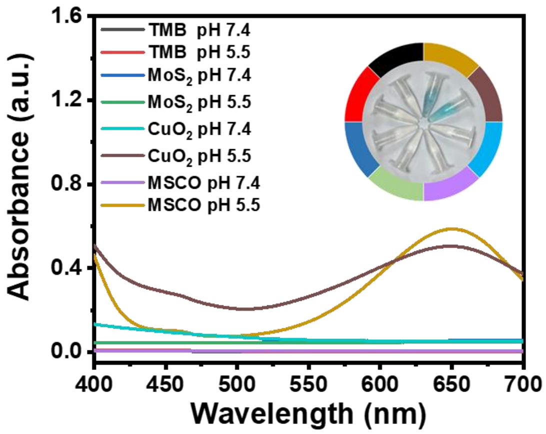

Figure S22. TMB oxidation ability of MoS_2, CuO_2 and MSCO under pH 7.4 and 5.5.

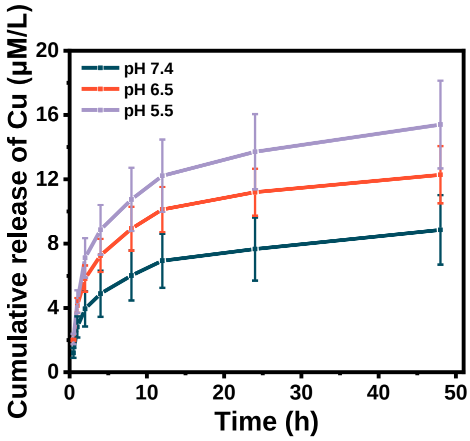

Figure S23 Cu^{2+} cumulative release of CAOP/M/PL hydrogel under different pH.

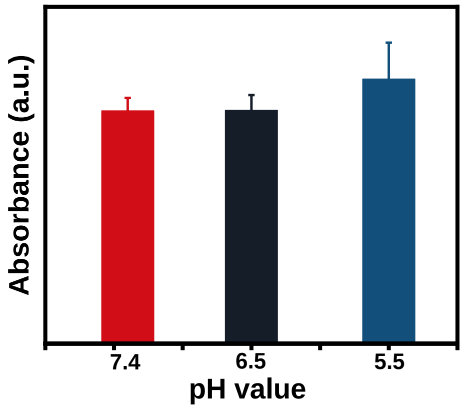

Figure S24. The change of GSH under different pH.

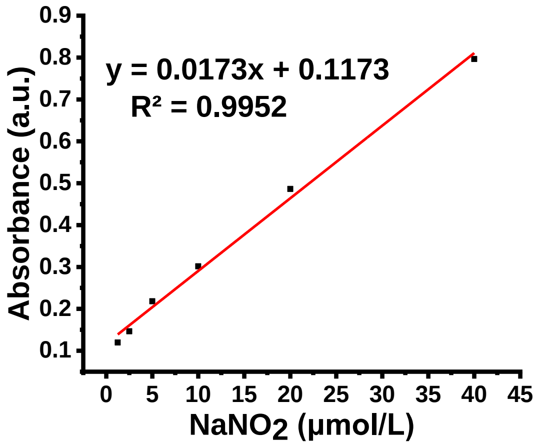

Figure S25. Standard curve of $NaNO_2$.

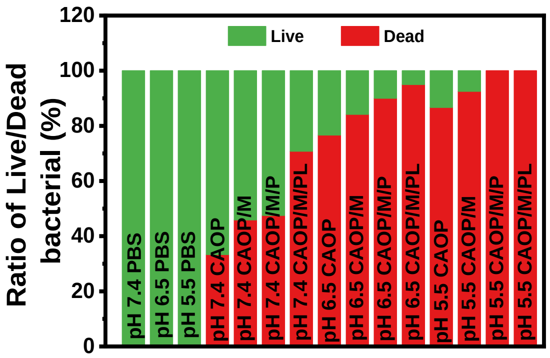

Figure S26. Ratio of live/dead bacterial after staining.

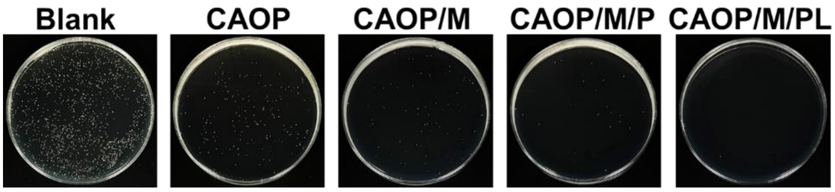

Figure S27. Presentation of in vivo antibacterial test.

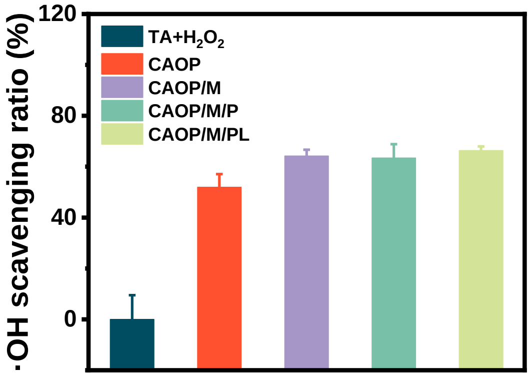

Figure S28. ·OH scavenging ratio of hydrogels.

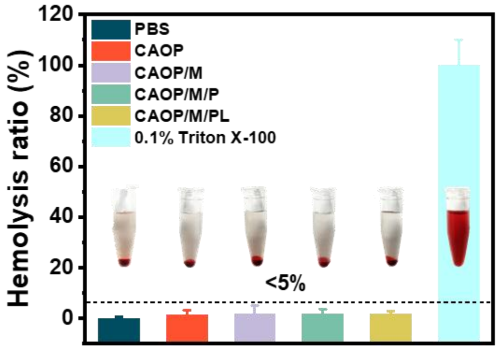

Figure S29. Hemolysis ratio of hydrogels.

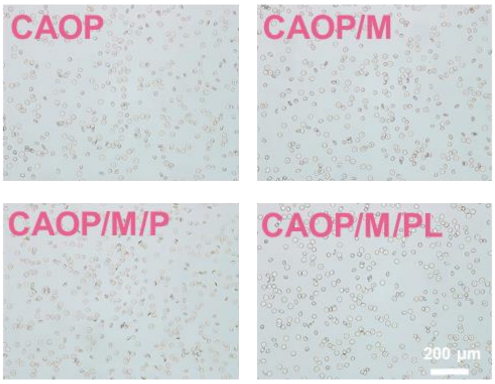

Figure S30. Image of erythrocytes under optical microscope.

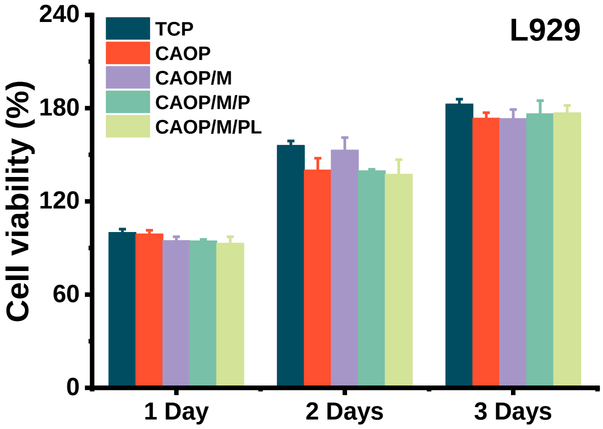

Figure S31. Biocompatibility test of hydrogels on L929 fibroblasts.

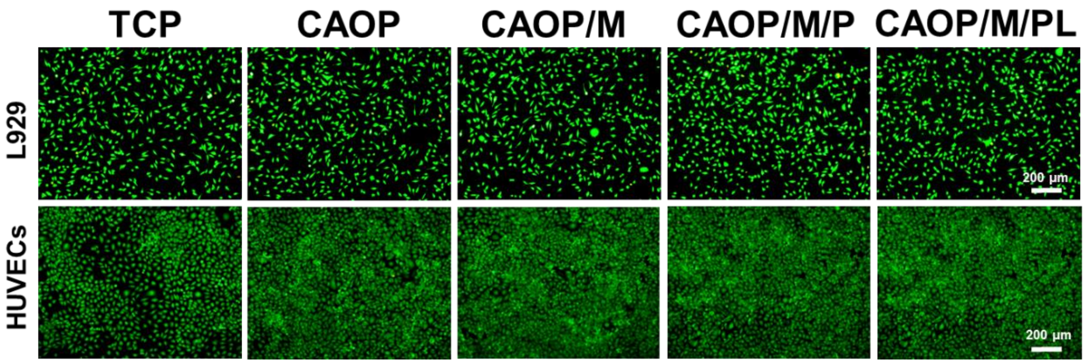

Figure S32. Live/dead staining of L929 fibroblasts and HUVECs.

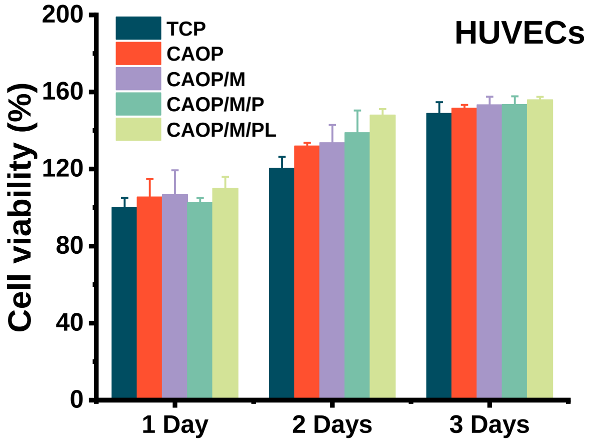

Figure S33. Proliferation test of HUVECs promoted by hydrogels.

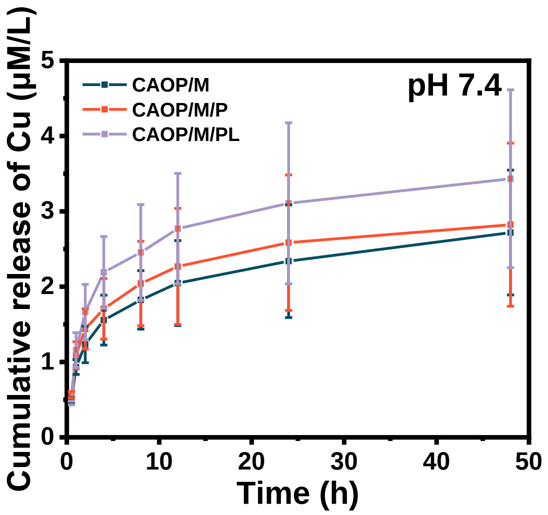

Figure S34. Cumulative release of Cu^{2+} under pH 7.4.

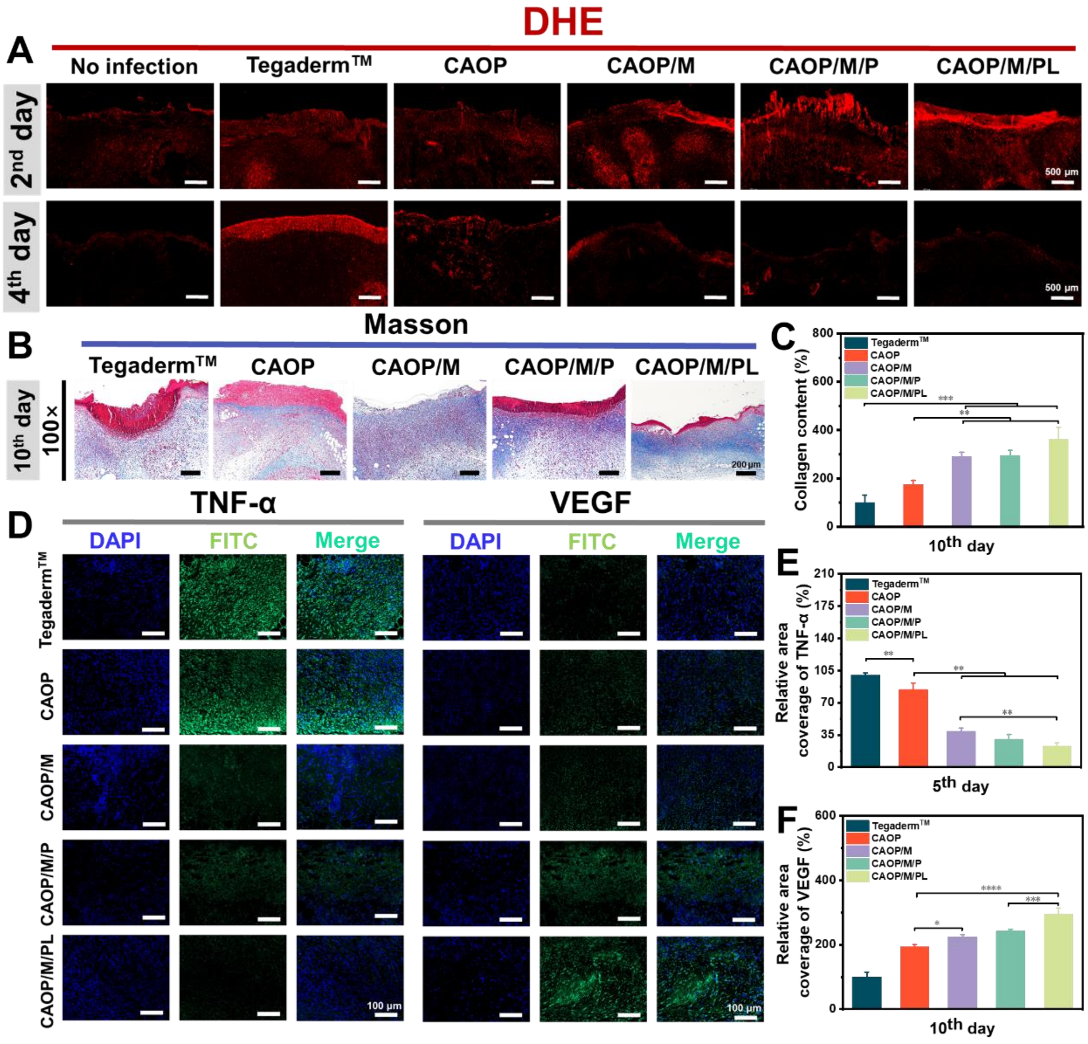

Figure S35. (A) DHE staining of the wound on the 2nd day and 4th day; (B) Masson staining of wound on 10th day; (C) Quantitative statistical results of collagen content on 10th day; (D) Expression of TNF-α on 5th day and VEGF on 10th day (green fluorescence); Quantitative statistical results of (E) TNF-α on 5th and (F) VEGF on 10th day. (*$p < 0.05$, **$p < 0.01$, ***$p < 0.001$, ****$p < 0.0001$)

REFERENCES

1. Chu L, Gao H, Cheng T *et al.* A charge-adaptive nanosystem for prolonged and enhanced in vivo antibiotic delivery. *Chem Commun*. 2016; **52**(37): 6265-6268. doi: https://doi.org/10.1039/C6CC01269H

2. Lin L-S, Huang T, Song J *et al.* Synthesis of Copper Peroxide Nanodots for H_2O_2 Self-Supplying Chemodynamic Therapy. *J Am Chem Soc*. 2019; **141**(25): 9937-9945. doi: https://doi.org/10.1021/jacs.9b03457

3. Song H, Wang Y, Fei Q *et al.* Cryopolymerization-enabled self-wrinkled polyaniline-based hydrogels for highly stretchable all-in-one supercapacitors. *Exploration*. 2022; **2**(4): 20220006. doi: https://doi.org/10.1002/EXP.20220006
